# Tropodithietic Acid, a Multifunctional Antimicrobial, Facilitates Adaption and Colonization of the Producer, Phaeobacter piscinae

**DOI:** 10.1128/msphere.00517-22

**Published:** 2023-01-09

**Authors:** Laura Louise Lindqvist, Scott A. Jarmusch, Eva C. Sonnenschein, Mikael Lenz Strube, Janie Kim, Maike Wennekers Nielsen, Paul J. Kempen, Erwin M. Schoof, Sheng-Da Zhang, Lone Gram

**Affiliations:** a Department of Biotechnology and Biomedicine, Technical University of Denmark, Kongens Lyngby, Denmark; b Department of Molecular Biology, Princeton University, Princeton, New Jersey, USA; c National Centre for Nano Fabrication and Characterization, Technical University of Denmark, Kongens Lyngby, Denmark; d Department of Health Technology, Technical University of Denmark, Kongens Lyngby, Denmark; e School of Biosciences, Geography, and Physics, Swansea University, Swansea, Wales, United Kingdom; Escola Paulista de Medicina/Universidade Federal de São Paulo

**Keywords:** biofilm, motility, *Phaeobacter*, secondary metabolites, tropodithietic acid, prophage, gene transfer agent, horizontal gene transfer, niche colonization

## Abstract

In the marine environment, surface-associated bacteria often produce an array of antimicrobial secondary metabolites, which have predominantly been perceived as competition molecules. However, they may also affect other hallmarks of surface-associated living, such as motility and biofilm formation. Here, we investigate the ecological significance of an antibiotic secondary metabolite, tropodithietic acid (TDA), in the producing bacterium, Phaeobacter piscinae S26. We constructed a markerless in-frame deletion mutant deficient in TDA biosynthesis, S26Δ*tdaB*. Molecular networking demonstrated that other chemical sulfur-containing features, likely related to TDA, were also altered in the secondary metabolome. We found several changes in the physiology of the TDA-deficient mutant, Δ*tdaB*, compared to the wild type. Growth of the two strains was similar; however, Δ*tdaB* cells were shorter and more motile. Transcriptome and proteome profiling revealed an increase in gene expression and protein abundance related to a type IV secretion system, and to a prophage, and a gene transfer agent in Δ*tdaB*. All these systems may contribute to horizontal gene transfer (HGT), which may facilitate adaptation to novel niches. We speculate that once a TDA-producing population has been established in a new niche, the accumulation of TDA acts as a signal of successful colonization, prompting a switch to a sessile lifestyle. This would lead to a decrease in motility and the rate of HGT, while filamentous cells could form the base of a biofilm. In addition, the antibiotic properties of TDA may inhibit invading competing microorganisms. This points to a role of TDA in coordinating colonization and adaptation.

**IMPORTANCE** Despite the broad clinical usage of microbial secondary metabolites with antibiotic activity, little is known about their role in natural microbiomes. Here, we studied the effect of production of the antibiotic tropodithietic acid (TDA) on the producing strain, Phaeobacter piscinae S26, a member of the *Roseobacter* group. We show that TDA affects several phenotypes of the producing strain, including motility, cell morphology, metal metabolism, and three horizontal gene transfer systems: a prophage, a type IV secretion system, and a gene transfer agent. Together, this indicates that TDA participates in coordinating the colonization process of the producer. TDA is thus an example of a multifunctional secondary metabolite that can mediate complex interactions in microbial communities. This work broadens our understanding of the ecological role that secondary metabolites have in microbial community dynamics.

## INTRODUCTION

Microorganisms often attach to and colonize biotic and abiotic surfaces, and such biofilms are believed to be the predominant microbial lifeform (1). In biofilms, multiple microbial species coexist in structured environments that allow for close interactions, including exchanging metabolites (2) and genetic material (3, 4). Additionally, surface-associated bacteria are more prolific producers of antimicrobial secondary metabolites (5–7) than their planktonic counterparts. These antimicrobial secondary metabolites are believed to provide a significant advantage to the producing microorganism by inhibiting competing microorganisms (8), in line with the classical perception that antimicrobial secondary metabolites predominantly act as competition molecules (5, 6). This perception has been challenged by the observation that many secondary metabolites engage in nonantibiotic activities, e.g., as signaling or nutrient-scavenging molecules (7, 9). Some of these nonantibiotic activities also affect hallmarks of surface-associated living, and traits such as biofilm formation and motility are often affected by sublethal concentrations of exogenous antibiotics (10–13).

Although it has been demonstrated that exogenous antibiotics affect bacterial phenotypes, only very few studies have explored how antibiotic production affects the producing organism itself. Recently, Zhang et al. (2021) showed that Photobacterium galatheae, which produces the antibiotic holomycin, is reduced in its biofilm-forming capacity when holomycin biosynthesis is abolished (14). Also, the lipopeptide surfactant facilitates horizontal gene transfer (HGT) in Bacillus subtilis by promoting cell lysis and DNA release (15) and may thereby facilitate niche adaptation (16, 17). Thus, production of molecules with antibiotic activity can affect the producing organism in previously unforeseen ways, some of which may provide more than one advantage to the organisms, for instance, during surface colonization. This indicates that there may be more than one reason secondary metabolite production prevails in the surface-associated microorganisms.

Tropodithietic acid (TDA) is a secondary metabolite produced by members of the globally occurring marine *Roseobacter* group (18–23), including the *Phaeobacter* genus, which exhibits strong adaptations for a surface-associated lifestyle (24, 25). In the closely related genus, *Tritonibacter*, TDA production and biofilm formation appear directly coupled since TDA is predominantly produced during stagnant (nonshaken) biofilm growth rather than during aerated planktonic growth (19, 26, 27). As an antibiotic, TDA targets both Gram-positive and Gram-negative bacteria through a proposed mode of action in which the proton motive force is disrupted (28). Introducing TDA and TDA-producing microorganisms into microbiomes reduces the abundance of closely related species and fast-growing secondary metabolite producers such as vibrios (29–32), indicating that TDA could arbitrate niche competition. In addition to its antibiotic properties, TDA is a weak chelator of iron, perhaps indicating a role as an iron reservoir (33). In Phaeobacter inhibens, exogenous TDA can substitute for N-3-hydroxydecanoylhomoserine lactone, a quorum sensing signal molecule, resulting in analogous changes in biofilm formation, motility, and antibiotic production (34–37). TDA production may be important in algal-*Phaeobacter* symbiosis as it has been suggested to protect the algae from bacterial pathogens (38, 39). TDA thus serves as a model molecule of a bacterial antibiotic secondary metabolite with multiple functions (21).

The purpose of the present study was to investigate the broader ecological roles of TDA in a producing bacterium, Phaeobacter piscinae strain S26, focusing on its possible involvement in surface colonization. To this end, we constructed a scarless TDA-deficient mutant, S26Δ*tdaB*, and compared the physiology of S26 wild type (WT) and mutant through a series of phenotypical assays, as well as global comparisons of the transcriptome, metabolome, and proteome. TDA serves as a case study for gaining a more holistic understanding of the ecological role of antibiotic secondary metabolites.

## RESULTS

### Deletion of *tdaB* abolishes TDA production and affects the expression of several TDA biosynthetic genes and proteins.

We generated a TDA-deficient mutant, Δ*tdaB*, by scarless deletion of the core biosynthetic gene *tdaB* of S26 WT (Fig. S1A and S1B). *tdaB* encodes a putative β-etherase/glutathione *S*-transferase, which is proposed to catalyze the addition of an *S*-thiocysteine to a CoA ester (40) in the biosynthetic pathway of TDA. To confirm the abolishment of TDA biosynthesis, Δ*tdaB* and WT were grown to stationary phase in marine broth (MB), and secondary metabolites were extracted and analyzed by HPLC-DAD-HRMS. TDA was detected in extracts of WT but not Δ*tdaB* (Fig. S1C). No precursors of TDA were found in WT or in Δ*tdaB*. TDA production of the mutant was partially restored upon genetic complementation with pBBR1MCS2_START-*tdaB* (Fig. S1C). The absence of TDA biosynthesis furthermore resulted in abolishment of antibiotic activity against V. anguillarum (Fig. S1D). TDA is an autoinducer of its own synthesis in some, but not all, TDA producing bacteria (23, 36), and we therefore inspected the expression of the remaining genes and proteins in the biosynthetic pathway (Fig. S1E). Genes *paaZ2*, *paaK2*, and *tdaF* were significantly less abundant in the mutant transcriptome, and the relative abundance of five proteins related to TDA biosynthesis was significantly lower in the Δ*tdaB* proteomic samples, including TdaC, TdaD, TdaR3, and Paaz2 in the culture supernatant proteome and TdaF in the cellular fraction.

10.1128/msphere.00517-22.5 FIG S1 (A) Genomic arrangement of the core biosynthetic pathway of TDA in Phaeobacter piscinae S26. The gene deleted in the TDA-deficient mutant is marked with an x. (B) PCR verification of S26Δ*tdaB*. Primers were designed to anneal up- and downstream of homology arms, and within *tdaB*. (C) Extracted ion chromatograms of *P. piscinae* S26 WT, Δ*tdaB*, and genetically complemented cultures showing the peak of TDA (m/z: 212.9683). (D) Well diffusion assay of Phaeobacter piscinae S26 WT, Δ*tdaB*, and genetic complements against V. anguillarum 90-11-287. (E) Transcriptome and proteome comparison of genes/proteins involved in TDA biosynthesis of samples that were differentially expressed in at least one comparison between *P. piscinae* S26 WT and Δ*tdaB* after 72 h of growth (stationary phase). T: Transcriptome. P-C: Proteome, cellular fraction. P-SN: Proteome, supernatant fraction. Download FIG S1, TIF file, 1.3 MB. Copyright © 2023 Lindqvist et al. 2023 Lindqvist et al. https://creativecommons.org/licenses/by/4.0/ This content is distributed under the terms of the Creative Commons Attribution 4.0 International license.

### Global transcriptomic and proteomic comparison of WT and the TDA-abolished mutant Δ*tdaB*.

The genome of S26 contains 4,077 open reading frames. Of the 4,004 genes detected (98.2% of all predicted genes) in the stationary-phase transcriptome, 519 were differentially expressed (*P* < 0.05, log2FC > 1, Fig. S2A) between WT and Δ*tdaB*. Of the 2,655 proteins detected from the proteome (65% of total predicted), 126 were differentially produced between WT and Δ*tdaB* (*P* < 0.01, log_2_ fold change > 1.5) in the cellular fraction (Fig. S2B), and 403 in the supernatant fraction (Fig. S2C). A list of log2FC and *P*-values can be found for both gene expression and protein abundances in Supplemental Data Set 1.

10.1128/msphere.00517-22.6 FIG S2 Volcano plots of proteome and transcriptome data from Phaeobacter piscinae S26 comparing S26 WT and Δ*tdaB*. Horizontal and vertical lines indicate significance thresholds. Blue: downregulated, red: upregulated, black: no difference. Genes were defined as significant at *P* > 0.05 and log2FC > 1. Proteins were defined as significant at *P* > 0.01 and log2FC > 1.5. (A) Transcriptome data. (B) Proteome, cell fraction. (C) Proteome, supernatant fraction. Download FIG S2, TIF file, 2.4 MB. Copyright © 2023 Lindqvist et al. 2023 Lindqvist et al. https://creativecommons.org/licenses/by/4.0/ This content is distributed under the terms of the Creative Commons Attribution 4.0 International license.

We then sought to link these transcriptomic and proteomic changes to the physiological changes. Following annotation with PROKKA, 1,903 proteins remained annotated as hypothetical proteins; further functional annotation using eggNOG-mapper reduced this number to 1,720 hypothetical proteins (Supplemental Data Set 1). Genes/proteins were divided into Clusters of Orthologous Groups (COGs) using eggNOG (Fig. S3). A large percentage yielded no hits (9.36%) or were categorized as unknown function (30.87%). Otherwise, the most affected COG group across all three sampling types was amino acid metabolism and transport which is also the most represented group across the genome, comprising 8.74% of all genes. In the transcriptome and supernatant proteome, a large fraction of downregulated genes/proteins in Δ*tdaB* compared to WT belonged to the energy production and conversion COG group.

10.1128/msphere.00517-22.7 FIG S3 Functional (COG) categories of differentially expressed genes and proteins in Phaeobacter piscinae Δ*tdaB* compared to S26 WT. Each color represents a comparison (transcriptomics, proteomics cell fraction, and proteomics supernatant fraction). Download FIG S3, TIF file, 2.3 MB. Copyright © 2023 Lindqvist et al. 2023 Lindqvist et al. https://creativecommons.org/licenses/by/4.0/ This content is distributed under the terms of the Creative Commons Attribution 4.0 International license.

### Deletion of *tdaB* alters the secondary metabolome of S26.

**FIG 1:**
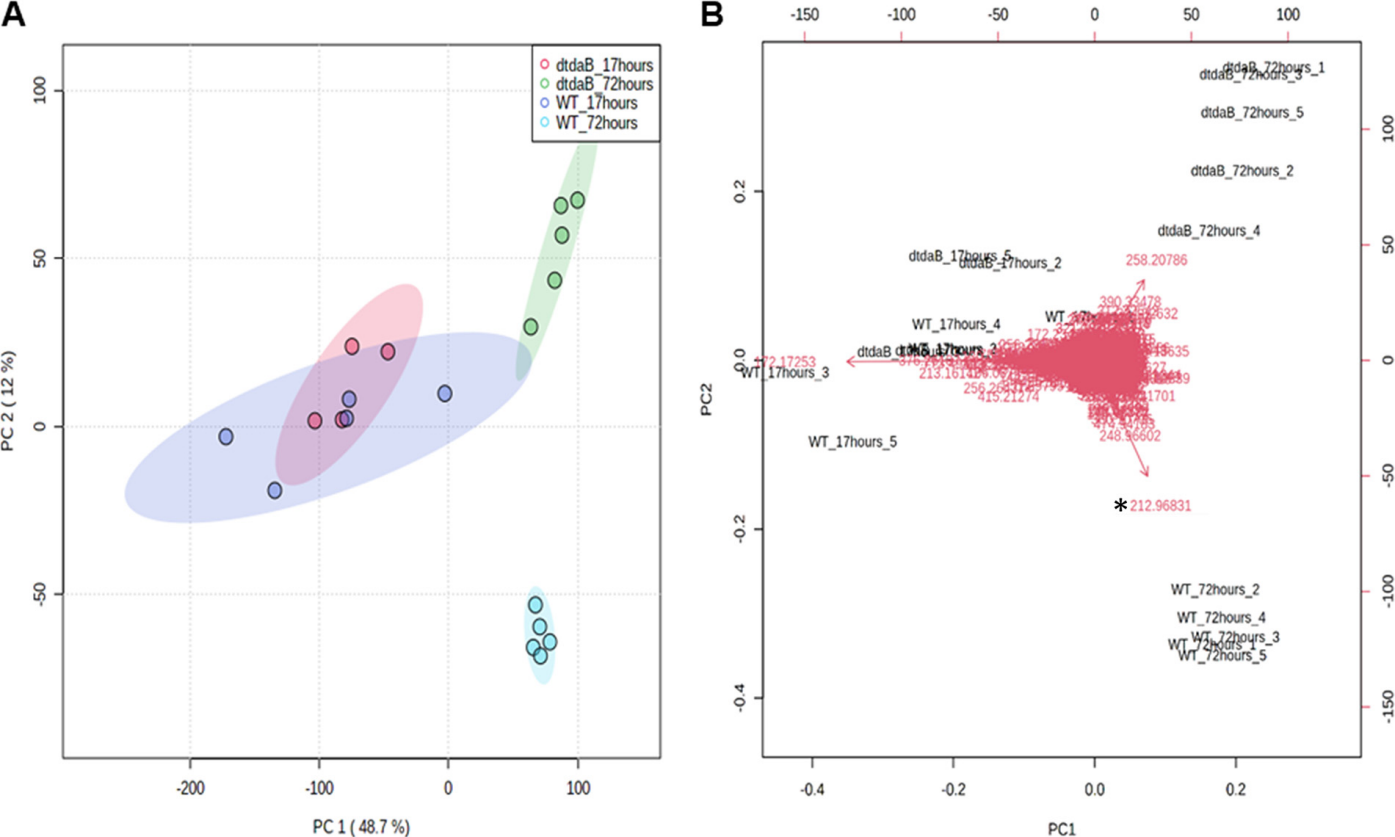
(A) Principal component analysis of metabolome of Phaeobacter piscinae S26 (WT) and Δ*tdaB* (dtdaB) mutant grown for 17 and 72 h (quintuplet replicates). Data were normalized to account for variable differences. Instrument and media blanks were included in this analysis but excluded from the PCA plot. (B) PCA biplot where samples (black) and features (red) are cocorrelated. Coordinate location of features corresponds to which samples they are found in. TDA (*m/z* 212.96831, indicated with a ‘*’) is located furthest from the origin and located in the same quadrant as S26-72 h samples.

Extracts of WT and Δ*tdaB* cultures were analyzed for changes in the secondary metabolome through LC-MS at two time points, 17 h (exponential phase) and 72 h (stationary phase). A principal-component analysis (PCA) showed marked convergence of WT and Δ*tdaB* at the exponential phase, whereas significant divergence was observed at the stationary phase (Fig. 1A). This divergence between WT and Δ*tdaB* cannot only be traced to the onset of significant TDA production (Fig. 1B and Fig. S4A) but many unknown features also drive this divergence (Fig. 1B and Supplemental Fig. S4A). All of this considered, the Δ*tdaB* mutant affects TDA-related metabolites as well as the secondary metabolome (typically masses above >300 Da [41]) beyond TDA).

10.1128/msphere.00517-22.8 FIG S4 (A) Correlation plot (generated using PCA analysis data) showing the 50 most significant features (represented as *m/z*) in a comparison between the metabolome of Phaeobacter piscinae S26 WT and Δ*tdaB* at 17 or 72 h along with their abundance for each individual sample. Samples are color-coded according to strain and sampling time point. Positive correlations (red boxes) represent features found in those samples whereas negative correlations (blue boxes) show the lack of detection. TDA clearly appears in the positively correlated features for the WT 72 h timepoint but so do many additional features. (B) Feature Based Molecular Network for LC-MS/MS data from Phaeobacter piscinae S26 WT and Δ*tdaB* grown for 17 (exponential phase) and 72 h (stationary phase). Nodes are distinguished by presence in S26 (lavender) or Δ*tdaB* mutant (green). The bar graphs on top of each node correspond to the timepoint tested (17 h (orange) or 72 h (blue)). Dashed connections (edges) correspond to networking via Ion Identity Molecular Networking and indicate features matched based on adduct searches, while solid connections correspond to traditional molecular networking based on fragmentation-matching. Download FIG S4, TIF file, 2.4 MB. Copyright © 2023 Lindqvist et al. 2023 Lindqvist et al. https://creativecommons.org/licenses/by/4.0/ This content is distributed under the terms of the Creative Commons Attribution 4.0 International license.

**FIG 2:**
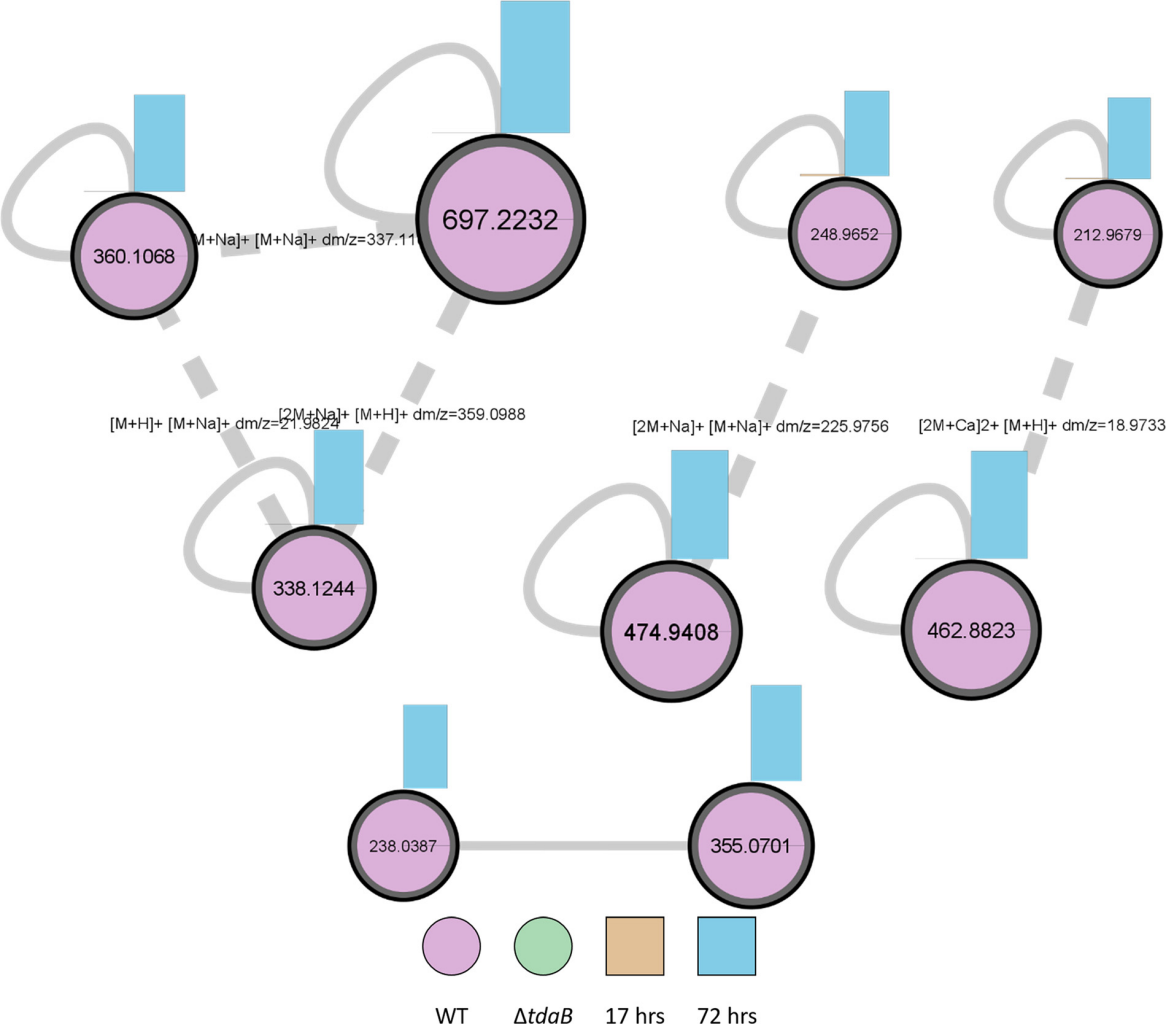
Molecular families corresponding to TDA (*m/z* 212.96), methyl-TDA (*m/z* 248.96) and a potential new TDA-biosynthesis derived feature (*m/z* 338.1244 and *m/z* 238.0387). Nodes are distinguished by presence in Phaeobacter piscinae S26 WT (pink) or Δ*tdaB* mutant (green). The bar graphs on top of each node corresponds to the time point tested (17 h (orange) or 72 h (blue)). Ion Identity Molecular Network allowed for connections to be made between the data (dotted edges), allowing for the annotation of TDA-related adducts and dimers. Traditional molecular networking represents these features as singletons and does not allow for these connections to be annotated easily.

We used feature-based molecular networking, Ion Identity molecular networking, and SIRIUS formula prediction to further explore the differences between WT and Δ*tdaB* (Fig. 2). Multiple small molecular families (groupings of related features) were observed only in WT samples at the stationary phase, including TDA and methyl troposulfenin (Fig. S4B). Additional features were observed within the same samples, possibly indicating a relationship to TDA production in the stationary phase. Ion identity molecular networking further indicated that many of these molecular families contain the monomeric unit and its accompanying dimer for both TDA and the other features (Fig. 2). To confirm the suspected relationship of these additional features to TDA, formula prediction was undertaken using SIRIUS. Overall, features *m/z* 338.1244 and *m/z* 238.0387 (Fig. 2) were only present in the stationary growth WT samples and contained sulfur in their predicted formulae (Supplemental Data Set 1, sheet “Formula predictions_SIRIUS_WT”).

### Cell morphology is altered in Δ*tdaB*.

**FIG 3:**
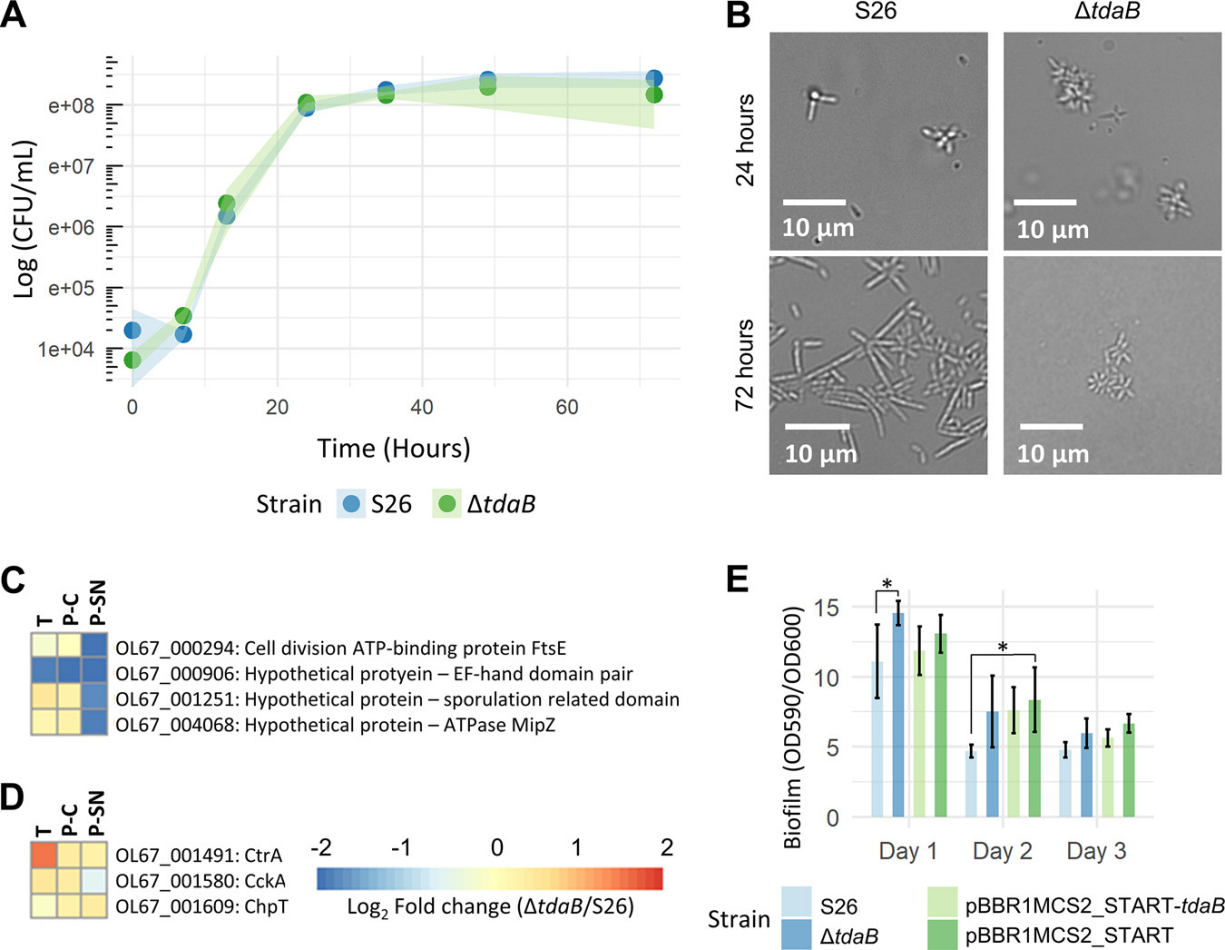
(A) Growth (log_10_[CFU/mL]) of Phaeobacter piscinae S26 WT and Δ*tdaB* over 72 h. Plot shows mean with standard deviation. (B) Cell morphology of S26 WT and Δ*tdaB* after 24 (end log-phase) and 72 h (stationary phase). (C) Log_2_(fold change) of proteins and genes belonging to COG D: Cell cycle control that were differentially expressed in at least one comparison out of three (transcriptome, cell-, or SN proteome) in Δ*tdaB* relative to S26 WT. T: Transcriptome. P-C: Proteome, cellular fraction. P-SN: Proteome, supernatant fraction. Same colorbar is used for both C and D. (D) Log_2_(fold change) of the *cckA*-*chpT*-*ctrA* phosphorelay system in Δ*tdaB* relative to S26 WT. (E) Biofilm formation of S26 WT, Δ*tdaB*, and genetic complements after one, two, and 3 days as assessed by a crystal violet assay. Statistical comparisons for each day were made through an ANOVA followed by a Tukey’s test. *, *P < *0.05; **, *P < *0.01; ***, *P < *0.001. Error bars indicate standard deviation.

The doubling times of WT and Δ*tdaB* were similar, being 1.04 ± 0.18 h and 0.93 ± 0.05 h (Student's *t* test, *P > *0.05, calculated based on log[CFU/mL] at 7 and 24 h), respectively (Fig. 3A). Both strains grew to a maximum cell density of 10^8^ CFU/mL, demonstrating that TDA production does not hinder nor enhance the growth of Phaeobacter piscinae S26. WT cells became elongated over time, with some cells reaching ~10 μM (Fig. 3B). Cell length varied and was on average 2.7 ± 2.2 μm after 3 days. Additionally, WT cells formed star-shaped rosettes. While Δ*tdaB* cells also formed rosettes, cell length was significantly reduced (Student's *t* test, *P < *0.001) to an average of 1.6 ± 0.3 μm with the longest observed cells reaching ~2 μM (Fig. 3B). Genetic complementation did not restore cell length after 3 days of incubation (data not shown); however, after 7 days of incubation genetically complemented cells reached an average cell length of 2.6 ± 1.0 μm while cells carrying an empty vector were significantly shorter (Student's *t* test, *P < *0.001), reaching an average cell length of 1.7 ± 0.6 μm (Fig. S5A). Similar cell morphology alterations were also observed in cultures of another TDA-producing bacterium, *P. inhibens* DSM17395, and its TDA-deficient mutant DSM17395 *tdaB*::*gmR* (Fig. S5B). We hypothesized these morphological changes may stem from changes in cell cycle control and therefore examined genes encoding proteins of the COG group D: Cell cycle control (Fig. 3C), but no obvious changes were observed as only one cell cycle-associated protein was significantly downregulated across all samples, OL67_000906, a hypothetical protein containing an EF-hand domain pair. We also investigated homologs of the CckA-ChpT-CckA phosphorelay system, as this has been linked to cell differentiation in other *Rhodobacteraceae* (42, 43) and found a significant upregulation of *ctrA* (log_2_[FC]: 1.6, *P < *0.001) in the transcriptome (Fig. 3D).

10.1128/msphere.00517-22.9 FIG S5 (A) Cell morphology of S26Δ*tdaB* carrying either pBBR1MCS2_START-*tdaB* or an empty vector control, pBBR1MCS2_START, after seven days of incubation. (B) Cell morphology of Phaeobacter inhibens DSM17395 WT and its corresponding TDA-deficient mutant, DSM17395 *tdaB*::*gmR*. (C) Transcriptome/proteome comparison between Phaeobacter piscinae S26Δ*tdab* and S26 WT of TonB iron-acquisition systems differentially expressed in at least one comparison. T: Transcriptome. P-C: Proteome, cellular fraction. P-SN: Proteome, supernatant fraction. Same colorbar is used for both C and D. (D) Transcriptome/proteome comparison between Phaeobacter piscinae S26Δ*tdab* and S26 WT of zinc-acquisition systems differentially expressed in at least one comparison. Download FIG S5, TIF file, 2.7 MB. Copyright © 2023 Lindqvist et al. 2023 Lindqvist et al. https://creativecommons.org/licenses/by/4.0/ This content is distributed under the terms of the Creative Commons Attribution 4.0 International license.

### Motility of Δ*tdaB* is increased compared to WT.

**FIG 4:**
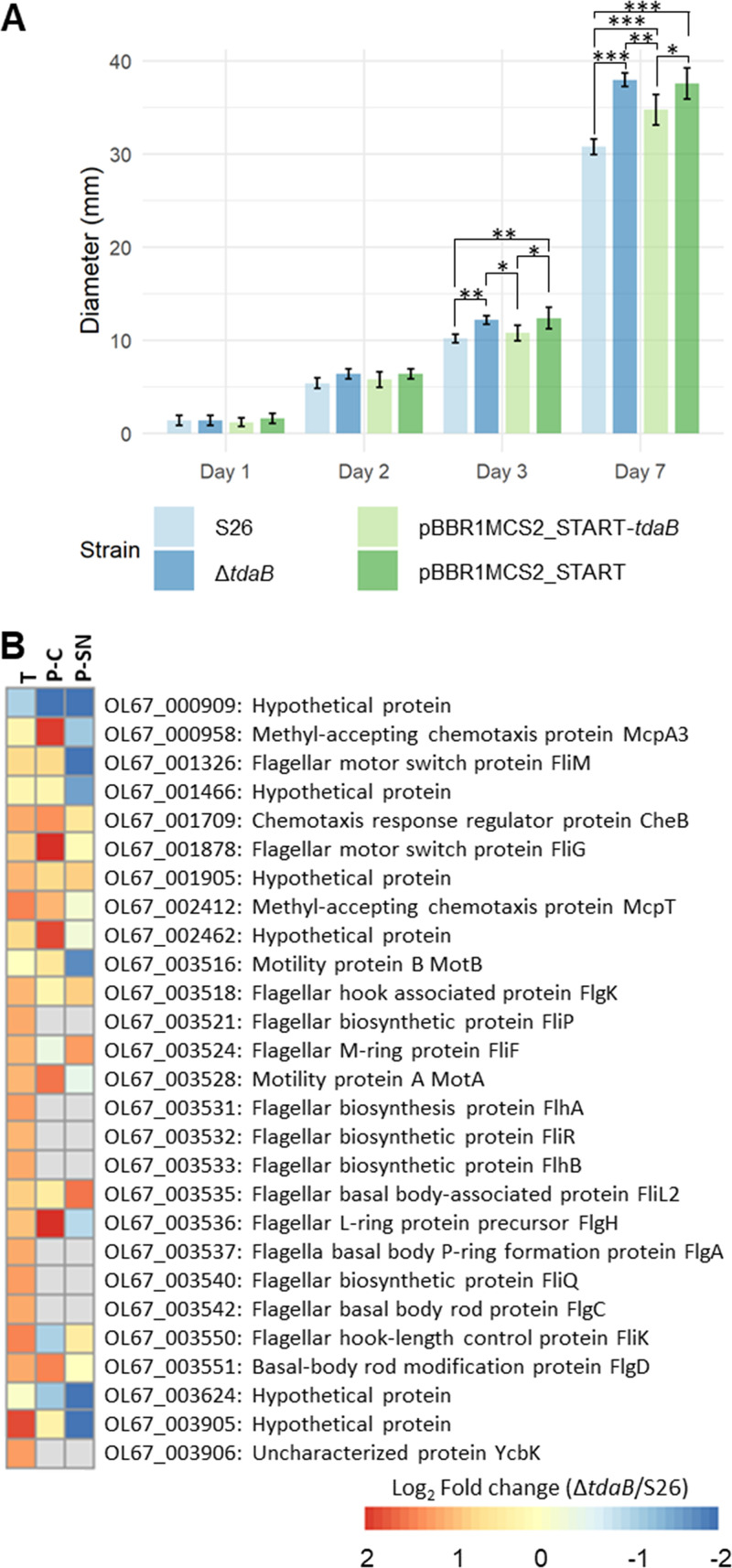
(A) Swimming motility of Phaeobacter piscinae S26 WT, Δ*tdaB*, and genetic complements. Statistical comparisons for each day were made through an ANOVA followed by a Tukey’s test. *, *P < *0.05; **, *P < *0.01; ***, *P < *0.001. Error bars indicate standard deviation. (B) Log_2_(fold change) of proteins and genes belonging to COG: N (Cell motility) differentially expressed in at least one comparison out of three (transcriptome, cell-, or SN proteome) in Δ*tdaB* relative to S26 WT. T: Transcriptome. P-C: Proteome, cellular fraction. P-SN: Proteome, supernatant fraction.

Since TDA can act as a QS-signaling molecule and previous studies have reported an effect of TDA production on biofilm formation and motility (34), we compared the capability of WT and mutant to form biofilm in a crystal violet assay biofilm after one, two, and 3 days (Fig. 3E). At day one, WT formed significantly less biofilm than Δ*tdaB*, and this difference was partially eliminated upon genetic complementation. However, on day two and three, no significant difference in biofilm formation was recorded between WT and Δ*tdaB* (Fig. 3E). The mutant Δ*tdaB* spread significantly faster than WT on soft agar after 3 days (Fig. 4A), and the diameter of the motility rings formed by Δ*tdaB* increased to 123.4 ± 2.3% of those formed by WT after 1 week. Reintroducing the *tdaB* gene to the mutant partially reduced the swimming of Δ*tdaB* to that of the WT, whereas introducing an empty control vector did not (Fig. 4A). Several genes or proteins involved in motility (COG: N, Fig. 4B), were upregulated in the mutant, including a region encoding the flagellar machinery (OL67_003516-3551). Three chemotaxis-related genes/proteins were also significantly upregulated in Δ*tdaB* in at least one comparison out of three (transcriptome, cell-, or SN proteome), i.e., a chemotaxis response regulator protein CheB (OL67_001709) and two methyl-accepting chemotaxis proteins OL67_000958 and OL67_002412.

### Metal metabolism is affected in Δ*tdaB*.

TDA is a weak iron-chelator (33), so we investigated whether iron-acquisition systems were affected in the transcriptome or proteome of Δ*tdaB*. The TonB system is involved in siderophore transportation (44); fittingly, the transcription of two predicted *tonB* genes, OL67_002475 and OL67_003315, were upregulated in the transcriptome of Δ*tdaB* compared to WT (Fig. S5C). In contrast, a transcript encoding a FurB-homologue (OL67_003754) that is part of the ferric uptake regulator family was less abundant in the mutant transcriptome. No evidence of metal chelation was observed in the metabolome. In the transcriptomic comparison, the most differentially expressed gene is OL67_002087, which is downregulated in the mutant and predicted to encode a homologue of the metal-binding protein ZinT (Fig. S5D). Because ZinT aids in zinc acquisition in Salmonella enterica during zinc shortage in conjunction with the ZnuABC system (45), we searched for any other changes in zinc metabolism. In the transcriptome, two genes (OL67_003813 and OL67_003815), encoding homologues of ZnuA and ZnuC, respectively, were downregulated in the mutant. Although not significantly downregulated in accordance with the set thresholds, OL67_002813 saw a (log_2_[FC]: 1.4, *p*: 0.03) lower relative abundance in the mutant cell proteome compared to WT.

### Several horizontal gene transfer (HGT) systems were highly expressed in Δ*tdaB*.

**FIG 5:**
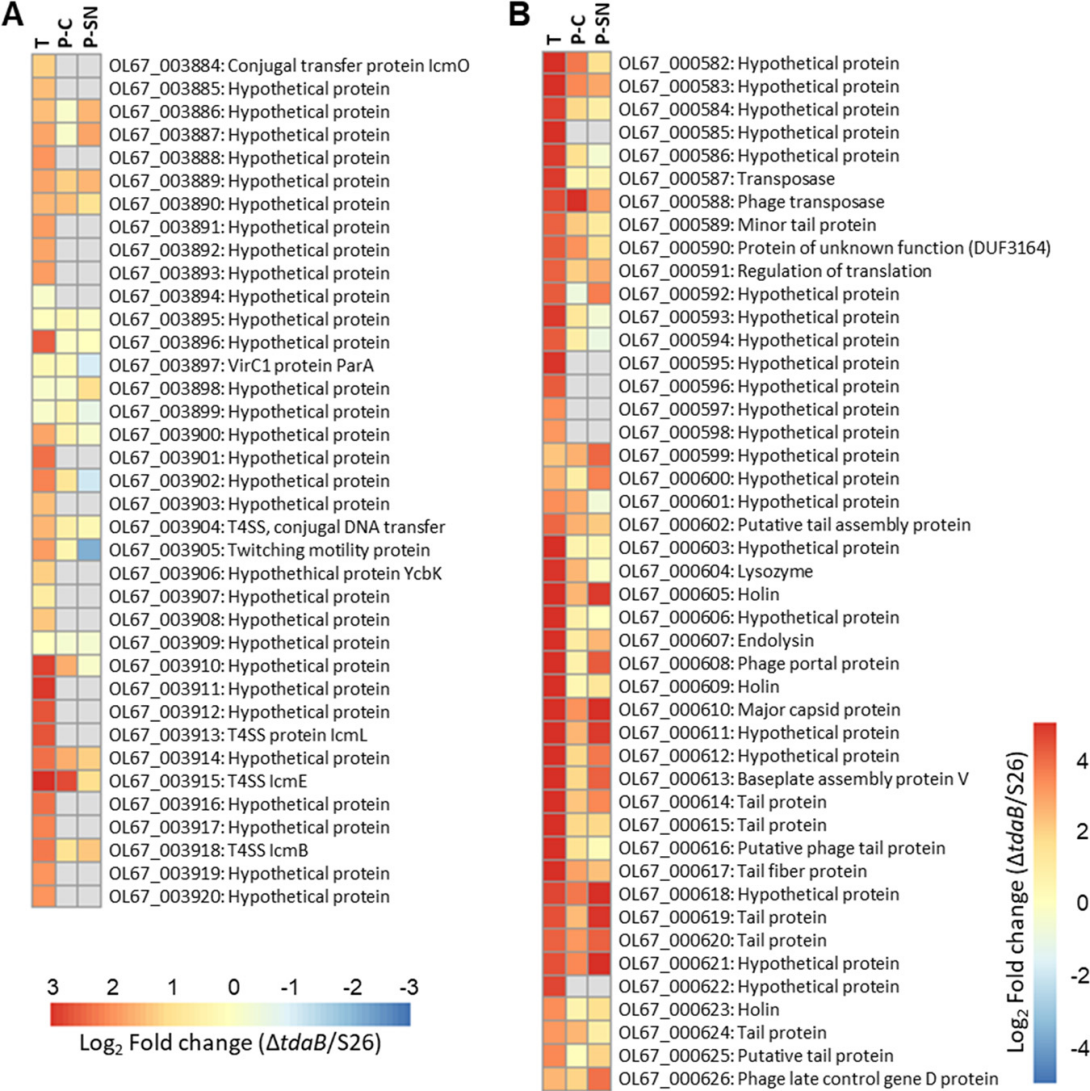
(A) Log_2_(fold change) of a region encoding a putative T4SS in Phaeobacter piscinae Δ*tdaB* relative to S26 WT. T: Transcriptome. P-C: Proteome, cellular fraction. P-SN: Proteome, supernatant fraction. (B) Log_2_(fold change) of a region encoding a putative prophage in Δ*tdaB* relative to S26 WT.

Both the transcriptome and proteome revealed a significant and systematic increase in expression of a region spanning from OL67_003884 to OL67_003920 (Fig. 5A) in Δ*tdaB* compared to WT. Although the region mainly encodes hypothetical proteins, several plasmid-located genes were predicted to encode proteins of a type IV secretion system (T4SS); OL67_002884, OL67_003913, OL67_003915, and OL67_003918 showed homology to proteins from the *icm* conjugal transfer system found in *Legionella* spp. (46). Notably, OL67_000327, predicted to encode a domain of a histone-like nucleoid-structuring (H-NS) protein HvrA, was the single most significantly downregulated protein (sorted by *P*-values) in the mutant for both cellular and supernatant proteome fractions (Supplemental Data Set 1). Histone-like nucleoid-structuring (H-NS) proteins are proposed to aid in regulation and physical integration following HGT (47).

**FIG 6:**
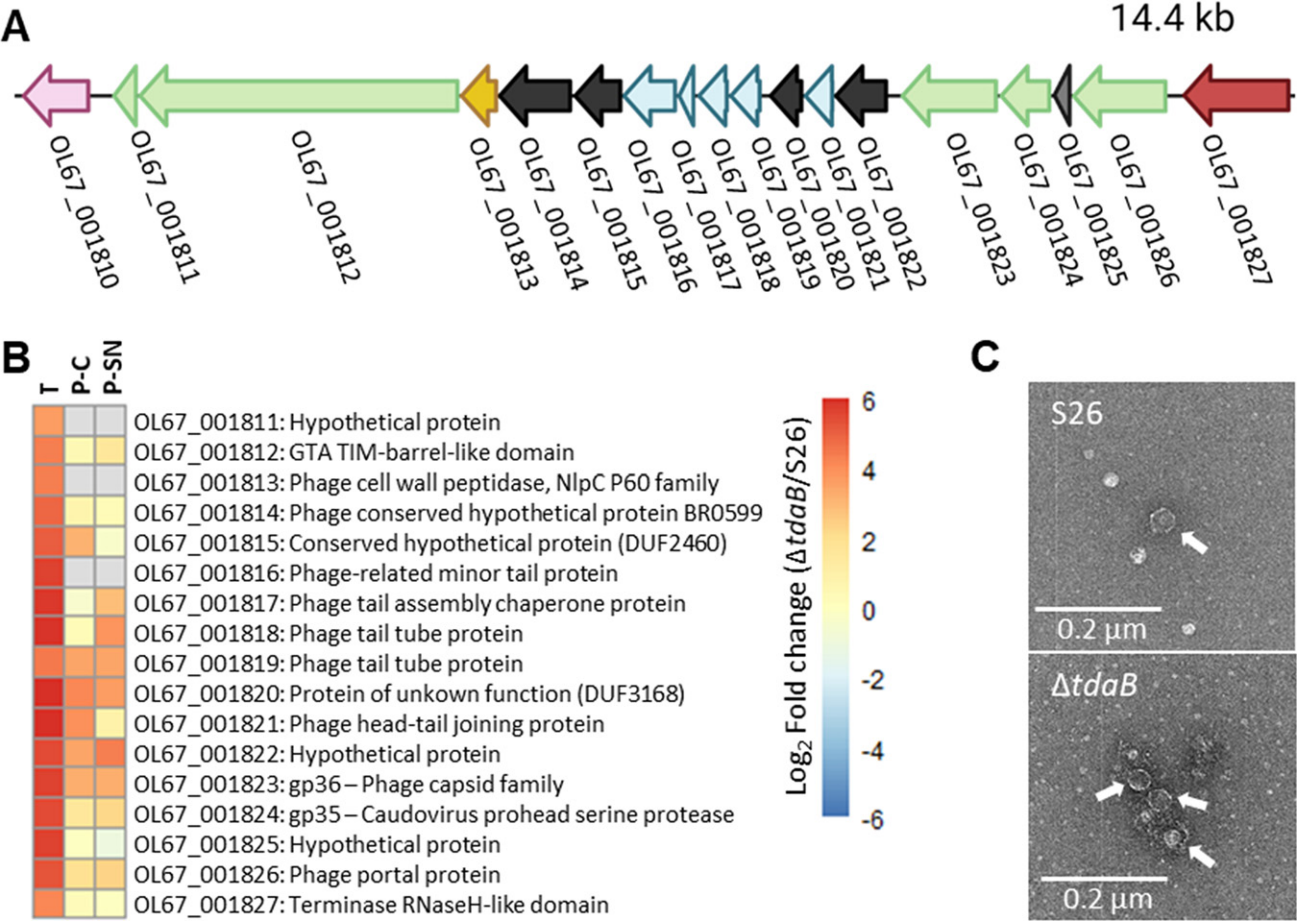
(A) Genomic arrangement of the predicted GTA in Phaeobacter piscinae S26. Pink, serine O-acetyltransferase (*cysE*); green, portal, capsid protease and major capsid proteins; yellow, lytic enzyme; black, unknown/hypothetical protein; blue, tail associated proteins; red, terminase large subunit. (B) Log_2_(fold change) of a region encoding a putative GTA in Δ*tdaB* relative to S26 WT. T: Transcriptome. P-C: Proteome, cellular fraction. P-SN: Proteome, supernatant fraction. (C) Transmission electron microscopy images of phage capsids (indicated by white arrows) in S26 WT and Δ*tdaB* supernatant.

Two other regions, OL67_000581:626 (Fig. 5B) and OL67_001810:27 (Fig. 6B), were also highly expressed/produced in the transcriptome and proteome of Δ*tdaB* (up to 40-fold) compared to WT. Both regions contained phage-associated proteins and were identified as a complete phage 1 and an incomplete phage 3, respectively, by PHAge Search Tool Enhanced Release (PHASTER, Table 1) (48, 49). Phage 1 (Table S1) consists of 46 proteins in total, with six proteins matching the Escherichia coli phage vB_EcoM-ep3. The differential expression of this region was not consistent across Δ*tdaB* replicates; in the transcriptome, this was observed in one out of three replicates, while in the proteome, three out of five replicates showed upregulation of the region (Fig. S6). Inspection of phage 3 revealed a genetic structure resembling a gene transfer agent (GTA, Fig. 6A and Table S1), as reviewed by Paul, 2008 (50). In contrast to phage 1, the GTA region was highly expressed/produced in all replicates of both transcriptome and proteome (Fig. 6B). PHASTER also detected two other prophages (Table 1), phage 2 and 4, which was not induced in the mutant cultures. Prophage- and GTA-release can be part of a stress response, and we therefore looked for changes in genes/proteins involved in the SOS response, but no differences were observed.

**TABLE 1:** PHASTER prediction. Four potential phage regions were identified in Phaeobacter piscinae S26 using PHASTER

Number	Region length	Completeness	Score	Number total proteins	Region position	Genome ID range	Most similar phage^*a*^	GC%
1	31.3 Kb	Intact	150	46	626399-657712	OL67_000581-626	PHAGE_Escher_vB_EcoM_ECO1230_10_NC_027995(6)	59.70%
2	44.2 Kb	Intact	150	38	692249-736465	OL67_000659-719	PHAGE_Escher_vB_EcoM_ECO1230_10_NC_027995(17)	58.71%
3	15.6 Kb	Incomplete	20	18	1889854-1905516	OL67_001810-1827	PHAGE_Roseob_1_NC_015466(4)	64.97%
4	30.6 Kb	Incomplete	50	43	2056836-2087483	OL67_001976-2018	PHAGE_Rhodov_vB_RhkS_P1_NC_031059(13)	59.75%

^*a*^The phage(s) with the highest number of proteins most similar to those in the region.

10.1128/msphere.00517-22.3 TABLE S1 PHASTER results for Phage 1 and Phage 3 (putative gene transfer agent) in Phaeobacter piscinae S26. Most similar BLAST hit for each protein is listed along with the corresponding E value. Download Table S1, DOCX file, 0.02 MB. Copyright © 2023 Lindqvist et al. 2023 Lindqvist et al. https://creativecommons.org/licenses/by/4.0/ This content is distributed under the terms of the Creative Commons Attribution 4.0 International license.

10.1128/msphere.00517-22.10 FIG S6 Gene/protein abundance in individual replicates for region encoding a putative prophage. Download FIG S6, TIF file, 2.4 MB. Copyright © 2023 Lindqvist et al. 2023 Lindqvist et al. https://creativecommons.org/licenses/by/4.0/ This content is distributed under the terms of the Creative Commons Attribution 4.0 International license.

To confirm the induction and subsequent release of prophages and/or GTAs, supernatants were visualized using transmission electron microscopy. Capsids were identified in both WT and Δ*tdaB* samples, indicating that TDA biosynthesis does not completely abolish phage and/or GTA release (Fig. 6C).

## DISCUSSION

Members of the *Phaeobacter* genus are predominantly surface-associated, and several species produce TDA, an antimicrobial secondary metabolite that provides the producers a competitive advantage by inhibiting competing bacteria in an ecological niche (24, 51). TDA may also act as a QS signal, affecting global gene expression (34). Here, we demonstrated that TDA biosynthesis, or rather the lack thereof, significantly changes the physiology of the producing strain, including several phenotypes associated with colonization of and adaption to novel niches.

Our results indicate that the secondary metabolism associated with TDA production drives significant changes in the producing organism, and that additional TDA-related metabolites proposed to derive from the same biosynthetic pathway (52), such as methyl-troposulfenin, show a similar reduction in the mutant. We also observed potential new chemical analogs that may derive from the TDA biosynthetic pathway. This confirms the previously observed promiscuity of the TDA biosynthetic pathway (53), resulting in the production of additional secondary metabolites beyond TDA. Such pathway promiscuity may result in analogs with individual functions as is, e.g., the case with surfactants where differences in fatty acid chain length dictate the regulatory outcome (54). Whether this could also be the case for the TDA biosynthetic pathway requires further studies.

Previous studies have reported that TDA-deficient mutants formed more biofilm and were hyper-motile compared to the WT and have proposed that this was linked to quorum sensing circuit(s) (34, 35, 55). We, similarly, observed an increase in motility of the mutant strains, further corroborated by an upregulation of flagellar proteins. We saw an initial increase in biofilm formation by Δ*tdaB* compared to WT, but this difference subsided after 2 days. However, the marked difference in cell morphology between WT and mutant could affect spatial localization in biofilms (56), but further studies are necessary to investigate these dynamics.

Several of our results point to a role of TDA production in repressing HGT, as proteins and genes belonging to a prophage, a GTA, and a T4SS located on one of the plasmids in S26 were highly expressed in the TDA-deficient mutant. Plasmid-located T4SS frequently mediate conjugation (57, 58), and prophages can similarly promote HGT through generalized transduction (59). GTAs are phage-like particles carrying pieces of host DNA found in the genomes of almost all marine *Rhodobacteraceae,* and these also mediate HGT (60–63). In *Phaeobacter* spp., which possess a high metabolic and ecological versatility (18, 64), GTAs are believed to be important drivers of diversification and niche adaptation (25). Hence, the impact of TDA production on these HGT systems may ultimately impact the genetic diversity of a population and, consequently, adaption to novel niches upon colonization, contributing to the widespread success of the *Roseobacter* group (65).

An obvious question for future studies is how TDA mediates these observed changes. As mentioned, TDA can act as a QS signal (32). QS-regulated flagellar motility (34) has been demonstrated in other *Phaeobacter* spp. and in other closely related *Rhodobacteraceae*, and QS has been linked to cell morphology changes (42, 66), T4SS expression (67), and GTA-release (42, 68–71). In several of these taxonomically close relatives, these phenotypical changes are mediated through the CtrA phosporelay system, which integrates QS inputs (42, 72, 73). We speculate that TDA may be incorporated as a QS signal in a similar system, as this would provide a unified explanation of the observed phenotypical changes, although further experimental evidence would be necessary to confirm this. Interestingly, this signaling cascade is involved in symbiont-relationships in two *Tritonibacter* spp., which can also be TDA-producing roseobacters (43, 73). It was recently suggested that up to one-third of TDA-producers are host-associated (74), and several studies have suggested a role for TDA in symbiotic interactions (38). In *T. mobilis*, TDA production increases in the presence of dimethylsulfoniopropionate (DMSP), an algal osmolyte (36), suggesting that TDA production is triggered in the presence of host-surfaces suitable for colonization.

Our results clearly show that TDA biosynthesis considerably changes the physiology of the producing organism. We suggest that TDA is not only an asset as part of a switch to a sessile lifestyle but is an integral signal controlling this switch. We, therefore, propose a model wherein TDA coordinates colonization behaviors, potentially through a QS-like mechanism. Planktonic cells will experience very low levels of TDA, leading to increased motility, allowing them to travel fast to novel niches and initiate colonization. During early colonization, high rates of HGT, mediated by conjugation, generalized transduction, and GTAs, are permitted by low TDA levels, which would allow for adaption to novel niches. Since prophage- and GTA-release comes with the trade-off cell lysis, tight control of this process is necessary to prevent a total collapse of the population: As the density of TDA producers increase, the accumulation of TDA halts the rate of HGT as a signal of successful adaptation and colonization. Simultaneously, the antimicrobial properties of TDA may ward off competing ecological neighbors, and TDA-mediated changes in motility and cell length may impact biofilm development, although the relationship between TDA production and biofilm remains somewhat ambiguous. Given the surface- and host-associated lifestyle of *Phaeobacter* spp., these results indicate that TDA may provide the producer with a competitive advantage in surface colonization in several ways. Hence, TDA becomes yet another example of the versatile nature of secondary metabolites and contributes to the building evidence that microbial secondary metabolites are more than “weapons of mass destruction.”

## MATERIALS AND METHODS

### Strains and culture conditions.

A list of strains used in this study can be found in Table S2. The *Phaeobacter* strains were cultured on Marine Agar (MA, BD Biosciences), in Marine Broth (MB, BD Biosciences), or Instant Ocean medium with Casamino Acids and glucose (IOCG, [51]) containing 30 g/L Instant ocean salts, (IO, Aquarium Systems, Inc.), 3 g/L HEPES (4-[2-hydroxyethyl]-1-piperazineethanesulfonic acid), 3 g/L Bacto Casamino Acids (BD Biosciences), and 2 g/L glucose adjusting pH was to 6.8–7.0. Cultures were grown at 25°C with shaking at 200 rpm, unless otherwise indicated.

10.1128/msphere.00517-22.4 TABLE S2 Primers, strains, and plasmids used for the study. Download Table S2, DOCX file, 0.03 MB. Copyright © 2023 Lindqvist et al. 2023 Lindqvist et al. https://creativecommons.org/licenses/by/4.0/ This content is distributed under the terms of the Creative Commons Attribution 4.0 International license.

10.1128/msphere.00517-22.5 DATA SET S1 Data from metabolomics, proteomics, and transcriptomics experiment. Download Data Set S1, XLSX file, 1.2 MB. Copyright © 2023 Lindqvist et al. 2023 Lindqvist et al. https://creativecommons.org/licenses/by/4.0/ This content is distributed under the terms of the Creative Commons Attribution 4.0 International license.

Escherichia coli strains (Table S2) were routinely cultured in LB broth (BD Biosciences) or LB agar (BD Biosciences) during conjugation. E. coli WM3064 used for electroporation was cultured in low salt LB broth (1 g/L NaCl, 5 g/L yeast extract, 10 g/L tryptone) or agar (1% wt/vol bacteriology grade agar) supplemented with 0.3 mM diaminopimelic acid (DAP). E. coli strains were grown at 37°C with shaking at 200 rpm. When required, antibiotics were added to the media in the following concentrations: 10 μg/mL (liquid) or 15 μg/mL (agar plates) chloramphenicol; 100 μg/mL (liquid) or 200 μg/mL (agar plates) ampicillin; 50 μg/mL (liquid and agar plates) kanamycin for E. coli strains; and 200 μg/mL (liquid and agar plates) kanamycin for *P. piscinae* mutant strains. For assays assessing the physiology of the strains, antibiotics were not added.

### Genome sequencing.

Genomic DNA was extracted using the Promega Wizard Genomic DNA purification kit (Promega) and Qiagen Genomic-tip 20/G. MinION sequencing (Oxford Nanopore Technologies) was performed using the Rapid Barcoding kit (SQK-RBK004) and the Flow Cell Priming kit (EXP-FLP002) following the protocol version RBK_9054_v2_revM_14Aug2019. Nanopore reads were demultiplexed using EPI2me (Oxford Nanopore Technologies). Adaptors were removed using porechop v0.2.4 (75). Oxford Nanopore MinION and Illumina MiSeq reads (obtained from a previous draft genome assembly project (NCBI accession number GCA_000826835.1) (76) were assembled using Unicycler v0.4.7 (77). The assembly was annotated using prokka v1.14.6 (78) and eggNOG-mapper V5.0 (79). To distinguish the hybrid assembly from the initial Illumina-based draft version of the genome on NCBI, the locus tags of the new annotation were adjusted to OL67XXXX.

### Construction and complementation of a scarless deletion mutant.

A list of plasmids used in the study can be found in Table S2. The suicide plasmid pDM4-d-*tdaB* was constructed and transferred to S26 by conjugation (Supplemental Materials 1). A two-step homologous recombination procedure based on sucrose-counterselection was used to generate the markerless *tdaB* gene in-frame deletion mutant, Δ*tdaB.* Genotype of mutants were verified with diagnostic PCR (Fig. S1B) and sequencing (Macrogen Europe) using oligoes in Table S2. For complementation, pBBR1MCS2_START-*tdaB* and the backbone vector pBBR1MCS2_START (80) were transferred to Δ*tdaB* through conjugation. Methods for DNA manipulation, plasmid construction and conjugation can be found in Supplemental Text S1.

10.1128/msphere.00517-22.5 TEXT S1 Supplemental Materials and Methods. Download Text S1, DOCX file, 0.1 MB. Copyright © 2023 Lindqvist et al. 2023 Lindqvist et al. https://creativecommons.org/licenses/by/4.0/ This content is distributed under the terms of the Creative Commons Attribution 4.0 International license.

### Global metabolome, transcriptome, and proteome experimental setup.

Strains were grown in MB and diluted 10,000-fold and grown stagnant in 5 mL IOCG in TPP6-well tissue culture plates (Merck) at 25°C. Plates were incubated in a humidity chamber to prevent desiccation. Samples were taken after 17 (exponential phase) and 72 h (stationary phase) for metabolome analysis, and after 72 h for transcriptomics and proteomics. Experiments were carried out with five biological replicates for metabolomics and proteomics, and in three biological replicates for transcriptomics. CFU determination and microscopy was performed for each sample. Transcriptomics, proteomics, and metabolomics workflows can be found in Supplemental Text S1.

### Antibiotic inhibition assays.

Vibrio anguillarum 90-11-287 (81) and the *Phaeobacter* strains were grown 3 days stagnant at 25°C in MB and *Vibrio* embedded in IO agar (30 g/L IO, 3.3 g/L Casamino Acids, 1% bacterial grade agar in dH_2_O). Sterile filtered (Minisart 0.2 μm filter (Merck) supernatant of *Phaeobacter* cultures were added to wells punched into the *Vibrio* agar and inhibition zones recorded after 3 days of incubation at 25°C.

### Growth kinetics.

*Phaeobacter* strains were grown in MB and diluted to OD_600_ = 0.00001 in IOCG and incubated at 25°C stagnant in TPP6-well tissue culture plates (Merck) with 5 mL media per well. The experiment was done in biological triplicates and growth followed by colony counts that were log transformed and plotted using ggplot2 (82) in Rstudio.

### Cell elongation assays.

*Phaeobacter* strains were grown in MB and diluted 10,000-fold and grown in IOCG in TPP6-well tissue culture plates (Merck) at 25°C in biological triplicates. Plates were incubated in a humidity chamber to prevent desiccation. For chemical complementation, samples were supplemented with 50% sterile-filtered WT or mutant supernatant of 3-day-old cultures grown in IOCG stagnant at 25°C. For genetic complementations, strains were grown in IOCG with 200 μg/mL kanamycin and incubated in the humidity chamber, stagnant at 25°C for 7 days. Cell morphology was assessed through brightfield microscopy using a Nikon ECLIPSE Ti2 inverted microscope at ×60 magnification. Cell length measurements were carried out using ImageJ (83). For each replicate, three pictures were taken and five random cells within each picture were measured for statistical comparisons.

### Swimming motility assays.

Swimming motility assays were performed in IOCG soft agar (0.3% wt/vol bacteriology grade agar). For supernatant complementation, plates were supplemented with 50% sterile-filtered supernatant of a 3 day old culture grown in IOCG stagnant at 25°C. Single colonies of strains to be tested were inoculated in MB and incubated stagnant for 2 days. A sterile needle was dipped in the tested cultures and used to inoculate the agar. Five biological replicates were included for each strain. For statistical comparison, a one way analysis of variance (ANOVA) followed by a Tukey’s test with a 95% confidence interval was carried out.

### Biofilm formation.

Biofilm formation was assessed using a modified protocol based on Jensen et al. (2007), Djordevic et al. (2002), and O’Toole and Kolter (1998) (84–86). Briefly, precultures were diluted 1,000-fold in IOCG and incubated in 96-well plates (ThermoFisher Scientific). MilliQ was added to border wells to prevent desiccation. Prior to staining, cell density was measured as OD_600_ in a SpectraMax i3 with 10 s orbital shaking prior to measurement. Wells were then washed with sterile MilliQ water and 125 μL crystal violet (CV, 1% wt/vol in sterile MilliQ) was added to each well and left for 15 min. CV was then removed, wells were washed three times with MilliQ, and the plate was set to dry in a flow bench for 15 min. 200 μL 96% EtOH was subsequently added to each well and left to sit for 30 min biofilm, and CV intensity was then measured at OD_590_ in a SpectraMax i3. Five biological replicates were used for each strain. For statistical comparison, a one way analysis of variance (ANOVA) followed by a Tukey’s test with a 95% confidence interval was carried out.

### Phage purification, transmission electron microscopy, and molecular detection.

Samples for transmission electron microscopy (TEM) were prepared according to Dragoš et al. 2021 (87). Bacterial supernatant was sterile filtered and pH adjusted to 7. PEG-solution (20% PEG-8000 [VWR International A/S], 116 g/L NaCl) was added in a 1:4 ratio, followed by incubation overnight at 4°C. After incubation, samples were centrifuged for 60 min at 18.500 g. Supernatant was then discarded and the pellet was resuspended in SM buffer (5.8 g/L NaCl, 0.96 g/L MgSO_4_, 6 g/L Tris-HCl, pH 7.5) to 1% of the initial volume. Five μL of purified phage sample were placed on freshly glow discharged Formvar coated 200 mesh nickel TEM grids (EMS Diasum) and allowed to adsorb for 5 min. Excess solution was wicked away with filter paper and the grids were subsequently rinsed 3 times on droplets of MilliQ water before being sequentially stained on droplets of 2% uranyl acetate in water for 10 s, 1 s, and 30 s, respectively. The excess staining solution was wicked away and the grids were dried in ambient conditions prior to imaging. Micrographs were acquired using a Tecnai T12 BioTwin TEM (Thermo Fisher Scientific) equipped with a Orius CCD camera (Gatan).

### Data availability.

The new version of the now closed Phaeobacter piscinae S26 genome with one chromosome and four plasmids is accessible on NCBI under the accession number CP080275-CP080279 (BioProject PRJNA266107). Metabolomics data were deposited and made publicly available in MassIVE at the following: MSV000089868. The mass spectrometry proteomics data have been deposited to the ProteomeXchange Consortium via the PRIDE partner repository with the data set identifier PXD035448. Bacterial strains are available upon request. RNAseq data have been uploaded to the Sequence Read Archive at NCBI under BioProject PRJNA859106.
