# Supplementary material for: Tropodithietic Acid, a Multifunctional Antimicrobial, Facilitates Adaption and Colonization of the Producer, Phaeobacter piscinae
Source: mSphere. 2023 Jan 9;8(1):e00517-22. doi: 10.1128/msphere.00517-22 (PMC9942592; doi:10.1128/msphere.00517-22)
Supplement: TABLE S1 [file msphere.00517-22-s0003.docx]

| **#** | **CDS Position** | | **BLAST Hit** | | | **E-Value** |
| --- | --- | --- | --- | --- | --- | --- |
|  | *Phage 1* | |  | | |  |
| 1 | complement(626399..627049) | | PHAGE_Pseudo_MP29_NC_011613: c repressor; OL67_000581; phage(gi215479977) | | | 9.35e-22 |
| 2 | 627161..627424 | | hypothetical protein; OL67_000582 | | | 0.0 |
| 3 | 627421..627645 | | hypothetical protein; OL67_000583 | | | 0.0 |
| 4 | 627642..627911 | | hypothetical protein; OL67_000584 | | | 0.0 |
| 5 | 627908..628192 | | PHAGE_Rhodob_RcapMu_NC_016165: hypothetical protein; OL67_000585; phage(gi356870849) | | | 1.08e-05 |
| 6 | 628202..628522 | | hypothetical protein; OL67_000586 | | | 0.0 |
| 7 | 628515..630779 | | PHAGE_Bacill_BalMu_1_NC_030945: hypothetical protein; OL67_000587; phage(gi100006) | | | 3.87e-10 |
| 8 | 630776..631501 | | PHAGE_Haemop_SuMu_NC_019455: phage transposase; OL67_000588; phage(gi418489040) | | | 8.40e-07 |
| 9 | 631498..631974 | | PHAGE_Xylell_Sano_NC_042344: minor tail protein; OL67_000589; phage(gi100056) | | | 7.03e-17 |
| 10 | 632027..632641 | | PHAGE_Burkho_KS10_NC_011216: hypothetical protein; OL67_000590; phage(gi198449283) | | | 5.94e-58 |
| 11 | 632692..632964 | | PHAGE_Bacill_SPbeta_NC_001884: histone-like prokaryotic DNA-binding protein family; OL67_000591; phage(gi9630187) | | | 1.33e-15 |
| 12 | 633021..633350 | | hypothetical protein; OL67_000592 | | | 0.0 |
| 13 | 633347..633595 | | hypothetical protein; OL67_000593 | | | 0.0 |
| 14 | 633645..634130 | | PHAGE_Faecal_FP_Mushu_NC_047913: hypothetical protein; OL67_000594; phage(gi100016) | | | 1.91e-07 |
| 15 | 634127..634330 | | hypothetical protein; OL67_000595 | | | 0.0 |
| 16 | 634317..634529 | | hypothetical protein; OL67_000596 | | | 0.0 |
| 17 | 634531..634869 | | hypothetical protein; OL67_000597 | | | 0.0 |
| 18 | 634878..635072 | | hypothetical protein; OL67_000598 | | | 0.0 |
| 19 | 635160..635426 | | PHAGE_Rueger_DSS3_P1_NC_025428: hypothetical protein; OL67_000599; phage(gi712912873) | | 4.14e-05 | |
| 20 | 635423..636340 | | PHAGE_Thermu_OH2_NC_021784: SPP1 family phage head morphogenesis protein; OL67_000600; phage(gi526118333) | | 1.25e-05 | |
| 21 | complement(636621..636824) | | hypothetical protein; OL67_000601 | | 0.0 | |
| 22 | complement(636824..637258) | | PHAGE_Pseudo_Lu11_NC_017972: putative tail assembly protein; OL67_000602; phage(gi388684690) | | 5.08e-09 | |
| 23 | complement(637271..637672) | | PHAGE_Rhizob_RR1_B_NC_021557: hypothetical protein; OL67_000603; phage(gi514231156) | | 4.62e-06 | |
| 24 | complement(637672..638502) | | PHAGE_Rhodob_RcCronus_NC_042049: hypothetical protein; OL67_000604; phage(gi100003) | | 1.21e-33 | |
| 25 | complement(638529..639155) | | PHAGE_Acinet_LZ35_NC_031117: holin; OL67_000605; phage(gi100034) | | 2.53e-07 | |
| 26 | complement(639198..639614) | | PHAGE_Strept_mu1/6_NC_007967: tail component protein; OL67_000606; phage(gi93007447) | | 3.58e-07 | |
| 27 | complement(639611..641347) | | PHAGE_Rhodov_vB_RhkS_P1_NC_031059: endolysin; OL67_000607; phage(gi100033) | | 8.15e-35 | |
| 28 | complement(641363..642643) | | PHAGE_Strept_Comrade_NC_048728: hypothetical protein; OL67_000608; phage(gi100025) | | 3.46e-17 | |
| 29 | 642800..643726 | | PHAGE_Lactoc_98201_NC_031064: holin; OL67_000609; phage(gi100034) | | 1.93e-20 | |
| 30 | 643723..644658 | | PHAGE_Strept_Jay2Jay_NC_029098: major capsid protein; OL67_000610; phage(gi985760260) | | 1.64e-05 | |
| 31 | 644662..645084 | | hypothetical protein; OL67_000611 | | | 0.0 |
| 32 | 645094..645600 | | hypothetical protein; OL67_000612 | | | 0.0 |
| 33 | 645597..646001 | | PHAGE_Ralsto_RSA1_NC_009382: baseplate assembly protein V; OL67_000613; phage(gi145708093) | | | 3.83e-14 |
| 34 | 646293..646646 | | PHAGE_Escher_vB_EcoM_ep3_NC_025430: tail protein; OL67_000614; phage(gi712913207) | | | 6.23e-35 |
| 35 | 646643..647536 | | PHAGE_Escher_vB_EcoM_ep3_NC_025430: tail protein; OL67_000615; phage(gi712913208) | | | 6.92e-79 |
| 36 | 647529..648188 | | PHAGE_Escher_vB_EcoM_ECO1230_10_NC_027995: putative phage tail protein; OL67_000616; phage(gi937533297) | | | 1.18e-29 |
| 37 | 648185..650242 | | PHAGE_Salmon_SEN5_NC_028701: tail fiber protein; OL67_000617; phage(gi966201574) | | | 4.31e-10 |
| 38 | 650229..650849 | | hypothetical protein; OL67_000618 | | | 0.0 |
| 39 | 650894..652063 | | PHAGE_Escher_vB_EcoM_ep3_NC_025430: tail protein; OL67_000619; phage(gi712913214) | | | 4.58e-103 |
| 40 | 652079..652585 | | PHAGE_Escher_vB_EcoM_ep3_NC_025430: tail protein; OL67_000620; phage(gi712913215) | | | 8.42e-30 |
| 41 | 652599..652892 | | hypothetical protein; OL67_000621 | | | 0.0 |
| 42 | 652889..653011 | | hypothetical protein; OL67_000622 | | | 0.0 |
| 43 | 653008..656085 | | PHAGE_Vibrio_1.202.O._10N.222.45.E8_NC_048066: holin; OL67_000623; phage(gi100034) | | | 2.29e-45 |
| 44 | 656078..656533 | | PHAGE_Escher_vB_EcoM_ep3_NC_025430: tail protein; OL67_000624; phage(gi712913218) | | | 1.29e-33 |
| 45 | 656530..656736 | | PHAGE_Vibrio_vB_VpaM_MAR_NC_019722: putative tail protein; OL67_000625; phage(gi428782752) | | | 4.13e-14 |
| 46 | 656741..657712 | | PHAGE_Escher_vB_EcoM_ep3_NC_025430: transcriptional regulator; OL67_000626; phage(gi712913220) | | | 7.05e-69 |
| *Phage 3 (Putative gene transfer agent)* | | | | | | |
| 1 | | complement(1889854..1890669) | | PHAGE_Strept_9873_NC_047763: hypothetical protein; OL67_001810; phage(gi100020) | 1.47e-06 | |
| 2 | | complement(1890937..1891257) | | hypothetical protein; OL67_001811 | 0.0 | |
| 3 | | complement(1891270..1895226) | | PHAGE_Rhodob_RcCronus_NC_042049: hypothetical protein; OL67_001812; phage(gi100019) | 0.0 | |
| 4 | | complement(1895226..1895696) | | PHAGE_Rhodob_RcCronus_NC_042049: hypothetical protein; OL67_001813; phage(gi100018) | 1.62e-16 | |
| 5 | | complement(1895693..1896643) | | PHAGE_Rhodob_RcCronus_NC_042049: hypothetical protein; OL67_001814; phage(gi100017) | 1.53e-36 | |
| 6 | | complement(1896643..1897275) | | PHAGE_Rhodob_RcCronus_NC_042049: hypothetical protein; OL67_001815; phage(gi100015) | 1.02e-55 | |
| 7 | | complement(1897291..1897947) | | PHAGE_Vibrio_SSP002_NC_041910: hypothetical protein; OL67_001816; phage(gi100042) | 7.06e-15 | |
| 8 | | complement(1897940..1898197) | | hypothetical protein; OL67_001817 | 0.0 | |
| 9 | | complement(1898194..1898583) | | hypothetical protein; OL67_001818 | 0.0 | |
| 10 | | complement(1898598..1899011) | | PHAGE_Rhodob_RcapNL_NC_020489: gene transfer aget (GTA) orfg9-like phage major tail protein; OL67_001819; phage(gi461474972) | 9.17e-22 | |
| 11 | | complement(1899117..1899530) | | hypothetical protein; OL67_001820 | 0.0 | |
| 12 | | complement(1899527..1899898) | | hypothetical protein; OL67_001821 | 0.0 | |
| 13 | | complement(1899895..1900542) | | PHAGE_Brucel_BiPBO1_NC_031264: hypothetical protein; OL67_001822; phage(gi100008) | 2.87e-05 | |
| 14 | | complement(1900729..1901913) | | PHAGE_Salmon_118970_sal3_NC_031940: hypothetical protein; OL67_001823; phage(gi100008) | 2.03e-71 | |
| 15 | | complement(1901962..1902582) | | PHAGE_Paraco_Shpa_NC_041868: hypothetical protein; OL67_001824; phage(gi100005) | 5.61e-42 | |
| 16 | | complement(1902615..1902833) | | hypothetical protein; OL67_001825 | 0.0 | |
| 17 | | complement(1902826..1904019) | | PHAGE_Gordon_Mahdia_NC_042089: hypothetical protein; OL67_001826; phage(gi100003) | 7.73e-24 | |
| 18 | | complement(1904224..1905516) | | PHAGE_Caulob_Sansa_NC_047756: hypothetical protein; OL67_001827; phage(gi100042) | 3.33e-88 | |
