# Supplementary material for: Tropodithietic Acid, a Multifunctional Antimicrobial, Facilitates Adaption and Colonization of the Producer, Phaeobacter piscinae
Source: mSphere. 2023 Jan 9;8(1):e00517-22. doi: 10.1128/msphere.00517-22 (PMC9942592; doi:10.1128/msphere.00517-22)
Supplement: TABLE S2 [file msphere.00517-22-s0004.docx]

| **Strain** | **Genotype** | **Reference** |
| --- | --- | --- |
| *Phaeobacter* *piscinae* S26 | Wild type | Grotkjær et al. 2016 (17) |
| *Phaeobacter* *piscinae* S26Δ*tdaB* | Δ*tdaB* | This study |
| *Phaeobacter* *inhibens* DSM17395 | Wild type | Ruiz-Ponte et al. 1998 (18) |
| *Phaeobacter* *inhibens* DSM17395 *tdaB::gmR* | *tdaB*::*gmR* | Wang *et al.* 2016 (19) |
| *Escherichia coli* WM3064 | *thrB1004 pro thi rpsL hsdS lacZ*Δ*M15 RP4-1360* Δ*(araBAD)567* Δ*dapA1341::[erm pir]* | Strain developed by William Metcalf at UIUC |
| *Escherichia coli* TOP10 | F- *mcrA* Δ*( mrr-hsdRMS-mcrBC)* Φ*80lacZ*Δ*M15* Δ*lacX74 recA1 araD139* Δ*(araleu)7697 galU galK rpsL (StrR) endA1 nupG* | ThermoFisher Scientific, C404010 |
| *Escherichia coli* GBdir-pir116 | F^-^ ∆*lac169 rpoS(Am) robA1 creC510 hsdR514 endA recA1 uidA(*∆*MluI)::pir-116* | Wang *et* *al*. (2016) (2) |
| *Vibrio anguillarum* 90-11-287 | Wild type | Skov *et al.* (1995) (20) |
| **Plasmid** | **Plasmid features** | **Reference** |
| pJET1.2 | pMB1 origin of replication, Amp^r^, P_lacUV5_, *eco47IR*, T7 promoter | ThermoFisher Scientific |
| pJET1.2-d-*tdaB* | pJET1.2 backbone carrying homology arms targetting *tdaB* | This study |
| pDM4 | *sacB*. *cat*. R6Kγ origin. | Milton *et al.* (1996) (21) |
| pDM4-d-*tdaB* | pDM4 backbone carrying homology arms targetting *tdaB* | This study |
| pBBR1MCS2_START | pBBR1 origin of replication, Kan^r^, P_lac_, *lacZα* | Obranić *et al.* (2013) (22) |
| pBBR1MCS2_START-*tdaB* | pBBR1MCS2_START backbone carrying *tdaB* | This study |
| **Primer** | **Sequence (5’-3’)** | **Description** |
| pDM4 Fw | CCAGAAGATGAAGCTGCTGGATGTCGACGGTATCGATAAGC | Amplification of pDM4 backbone |
| pDM4 Rv | GTTCTTCGACAGATCTGCCAGGATCTTGCATGCGGGTAAC |  |
| L arm Fw | GTTACCCGCATGCAAGATCCTGGCAGATCTGTCGAAGAAC | Amplification of 5' homology arm |
| L-arm Rv | AATCTCACTAAGGAGCGCACACCTCTGACTATCTTGCGTG |  |
| R-arm Fw | TGACACGCAAGATAGTCAGAGGTGTGCGCTCCTTAGTGAG | Amplification of 3' homology arm |
| R arm Rv | GCTTATCGATACCGTCGACATCCAGCAGCTTCATCTTCTGG |  |
| DtdaB P1 | CAACCTGGGATCAACTGAGG | Primers for checking homologous crossovers |
| DtdaB P2 | ACATAGGCTGTACAGCTTGC |  |
| DtdaB P3 | GATGGCGTTGGTCGGAATC |  |
| DtdaB P4 | AGGACCATATTGTCGGTGTTC |  |
| CmR Fw | GGCATTTCAGTCAGTTGCTC |  |
| CmR Rv | CCATCACAAACGGCATGATG |  |
| tdaB fw | TCACACAGGAAACACATATGAACCGCAAGCTGTACAGC | Amplification of *tdaB* gene |
| tdaB Rv | TCACTATAGGGCGAATTGTCAGCCTGACTGGGCCGGATTC |  |
| pBBR fw | GAATCCGGCCCAGTCAGGCTGACAATTCGCCCTATAGTGAG | Amplification of pBBR1MCS2_START backbone |
| pBBR Rv | AGGCTGTACAGCTTGCGGTTCATATGTGTTTCCTGTGTG |  |
| Seq DtdaB P1 | CGATCTGCTGAATACCGAAC | Sequencing primers, S26Δ*tdaB* |
| Seq DtdaB P2 | CATCACAGGTTCCAACGAATC |  |
| Seq P1 Fw | GAGTTAGCTCACTCATTAGG | Sequencing, complementation plasmid |
| Seq P2 Rv | GCTTCCGGTAGTCAATAAAC |  |
| pDM4-d-tdaB seq P1 | TTCTTGTCTAGGTTTGCACC | Sequencing, pDM4-d-tdaB |
| pDM4-d-tdaB seq P2 | AGTCTCTGGGTGTGATTGC |  |
| pDM4-d-tdaB seq P3 | AGTCAAGTTCTGTGCGTTCC |  |
