# Supplementary material for: Tropodithietic Acid, a Multifunctional Antimicrobial, Facilitates Adaption and Colonization of the Producer, Phaeobacter piscinae
Source: mSphere. 2023 Jan 9;8(1):e00517-22. doi: 10.1128/msphere.00517-22 (PMC9942592; doi:10.1128/msphere.00517-22)
Supplement: TEXT S1 [file msphere.00517-22-s0002.docx]

Supplemental Methods:

**Tropodithietic acid, a multifunctional antimicrobial, facilitates adaption and colonization of the producer, *Phaeobacter piscinae***

Laura Louise Lindqvist^1^, Scott A. Jarmusch^1^, Eva C. Sonnenschein^1, 5^, Mikael Lenz Strube^1^, Janie Kim^1,2^, Maike Wennekers Nielsen^1^, Paul J. Kempen^3,4^, Erwin M. Schoof^1^, Sheng-Da Zhang^1^ & Lone Gram^1^*

1. Department of Biotechnology and Biomedicine, Technical University of Denmark, 2800 Kgs. Lyngby, Denmark

2. Department of Molecular Biology, Princeton University, Princeton, NJ 08544, USA.

3. National Centre for Nano Fabrication and Characterization, Technical University of Denmark, 2800 Kgs. Lyngby, Denmark

4. Department of Health Technology, Technical University of Denmark, 2800 Kgs. Lyngby, Denmark

5. School of Biosciences, Geography, and Physics, Swansea University, Singleton Park, Swansea SA2 8PP, Wales, United Kingdom

*Corresponding author: [gram@bio.dtu.dk](mailto:gram@bio.dtu.dk)

**Keywords**

Biofilm, motility, *Phaeobacter*, secondary metabolites, tropodithietic acid, prophage, gene transfer agent, horizontal gene transfer, niche colonization

**Supplementary materials and methods**

**DNA manipulation.** Plasmids and primers used in the study are listed in **Supplemental table S2**. Genomic DNA extractions were performed using the NucleoSpin® Tissue kit (Macherey-Nagel, Fisher Scientific). Primers were synthesized by Integrated DNA Technologies (Belgium). Amplification of fragments for cloning were performed using Q5® High-Fidelity 2X Master Mix (NEB, Bionordika, Denmark), whilst diagnostic PCRs were performed with TEMPase Hot Start 2X Master Mix K (Ampliqon, VWR). PCR products were purified with the GFX^TM^ PCR DNA and Gel Band Purification Kit (GE Healthcare). The Monarch^®^ Plasmid Miniprep Kit (NEB, Bionordika) was used for plasmid extractions. Restriction enzymes were acquired from New England Biolabs (NEB, Bionordika).

**Plasmid construction.** Plasmids were designed in ApE - A plasmid Editor (v2.0) (1)and assembled through Direct RecET-mediated cloning as described by Wang *et al.* (2016) (2). For knocking out *tdaB*, homology arms of 1 kb flanking *tdaB* were amplified using primers L-arm Fw/Rv and R-arm Fw/Rv (**Supplemental table S2**) and merged through overlap PCR. The subsequent homology arms were introduced into pJET1.2 using the CloneJET PCR Cloning Kit (ThermoFisher Scientific) and transferred to *E. coli* TOP10 (Invitrogen). Primer pairs L-arm Fw/R-arm Rv and pDM4 Fw/Rv were used to amplify the homology arms and the linearized suicide vector pDM4, respectively. The resulting products were merged to plasmid pDM4-d-*tdaB* using direct cloning (2). For the complementation, the *tdaB* gene and the vector backbone were amplified using primers tdaB Fw/tdaB Rv and pBBR Fw/pBBR Rv, respectively and assembled to form pBBR1MCS2_START-*tdaB*. The recombinant plasmids were verified by restriction digestion and sequencing (Macrogen Europe).

**Bacterial conjugation.** Bacterial conjugation was performed according to the method described by Zhang *et al.* 2021 (3) with few modifications. In brief, the plasmids were transferred into *E. coli* WM3064 by electroporation and the recombinants were used as the donors in conjugation. Overnight pre-cultures of donors and recipients were diluted 100-fold and grown until mid-exponential phase. Donors were collected through centrifugation and washed once with LB + DAP to remove antibiotics. Recipients were added to the donors in a volume ratio of 1:6, pelleted via centrifugation, suspended in LB + DAP and mated on sterile 0.2 µM pore-sized mixed cellulose esters (MCE) membranes (Merck) on LBA + DAP plates for 3-4 hours at 30°C. After mating, the cells were recovered in MB for 60 minutes with aeration. Subsequently, the cells were plated with appropriate antibiotics and incubated at 25°C until colonies appeared.

**Transcriptomics preparation and analysis.** Three 500 µl aliquots of each sample were each added to 1 ml RNAprotect Bacteria Reagent (Qiagen), vortexed for 5 seconds, incubated at room temperature for 5 minutes, and centrifuged at 5,000 x g for 10 minutes. The samples were then decanted and residual supernatant was removed by dabbing the lip of the tubes onto a paper towel. The pelleted cells were mixed with 200 µl TE buffer (10 mM Tris-Cl, 1 mM EDTA, pH 8.0) containing 1 mg/ml lysozyme by vortexing, and then incubated at room temperature for 10 minutes, vortexing for 10 seconds every 2 minutes. 700 µl RLT buffer containing 1% β-mercaptoethanol was added and vortexed; in order to remove resulting residual particles, samples were centrifuged at 10,000 x g for 2 minutes and the supernatant was transferred into clean tubes. 500 µl ≥99.8% Ethanol BioUltra (Fluka Analytical) was added to each lysate and mixed by pipetting. Total RNA was then extracted using the RNeasy Mini Kit (Qiagen 74104) and the manufacturer-provided Protocol 7, with the following specifications: the three aliquots for each sample were pooled pre-extraction, centrifuging steps were performed at 10,000 x g, and the final elution was performed with 50 µl RNAse-free water. The resulting crude RNA was quantified using the Qubit^TM^ 2.0 RNA High Sensitivity Assay Kit (ThermoFisher Scientific), and then DNase-treated with the TURBO DNA-*free*^TM^ Kit (Invitrogen) following the manufacturer’s protocol for routine DNase treatment. The purified RNA was quantified again with the Qubit^TM^ 2.0 RNA High Sensitivity Assay Kit and stored at -80 °C. Prior to sequencing, the integrity of the RNA was assessed using Agilent RNA 6000 Nano LabChips (Agilent Technologies) run in an Agilent 2100 Bioanalyzer. 1 µg of each RNA sample was sent to Novogene for processing, library preparation, and directional RNA-sequencing (NovaSeq).

The resulting sequencing files were mapped to the annotated genome described above with Rsubread-package (4) using default settings. The feature count table was then analyzed with a negative binomial model and the Wald test was used to test significant differences between WT and Δ*tdaB* samples using DESeq2 (5). Genes were considered differentially expressed when adjusted p-values were <0.05 and log2 fold changes were >1.

**Proteomics sample preparation.** 5 mL sample was harvested at 5,000 x g for 5 min, and the supernatant was transferred to a 15 mL falcon tube. The cell fraction was washed twice with 1 mL ice-cold PBS buffer. From here on, Eppendorf Protein LoBind tubes were used. Between washing steps, cells were harvested at 7,000 x g for 1 min and PBS buffer was carefully removed. Lysis buffer (6M Guanidinium Hydrochloride, 10mM tris-(2-carboxy-ethyl)-phosphine, 40mM 2-Chloroacetamide (CAA), 50mM HEPES, pH8.5) was added and samples were incubated at 95°C for 5 min and subsequently cooled on ice. Ice-cold acetone was added to the supernatant to a final percentage of 80% and stored overnight at -20°C to precipitate protein. Proteins were precipitated at 2,000 x g for 20 min, acetone carefully removed and the pellet dried in a fumehood. The protein pellet was resuspended in lysis buffer and proteins from supernatant and cell samples were treated identically using a modified version of the protocol described by Schoof *et al.*, 2016 (6). Samples were sonicated for 5 x 30 s using BioRuptor on maximal setting, harvested and protein concentrations determined using BCA gold. 10 µg sample were diluted in 20 µL lysis buffer. Samples were diluted 3-fold with digestion buffer (10% acetonictrile (ACN), 50 mM HEPES pH 8.5) and LysC was added in a 1:50 ratio, and the sample was incubated for 3 hrs at 37°C. Sample were diluted 10-fold with digestion buffer relative to the starting volume, trypsin was added in a 1:100 ratio and samples were incubated overnight at 37°C. Samples were diluted with 2% trifluoroacetic acid (TFA) to a final percentage of 1% TFA. For each sample, a stage tip was prepared by adding three C18 discs to a 200 µL pipette tip. Each stage tip was activated with 20 µL 100% MeOH, 20 µL Buffer B (80% CAN, 0.1% formic acid (FA)), and 2 x 20 µL Buffer A’ (3% ACN, 1% TFA). Sample was then loaded onto the stage tip, 50 µL at a time. Filters were washed with 2 x Buffer A (0.1% FA in MiliQ water) and samples were subsequently eluted with Buffer B’ (40% ACN, 0.1% FA). Buffer was then evaporated in a Speedvac 5301 (Eppendorf) for 60 minutes at 60°C and peptides were resuspended in Buffer A* (2% ACN, 1% TFA). Concentration was determined using a DeNovix ds-11 fx+ Spectrophotometer.

**Proteome (Mass Spectrometry) Analysis.** For each sample, 500 ng of peptide was loaded onto a 2cm C18 trap column (ThermoFisher Scientific), connected in-line to a 50cm C18 reverse-phase analytical column (Thermo EasySpray ES803) using 100% Buffer A (0.1% Formic acid in water) at 750bar, using the Thermo EasyLC 1000 HPLC system, and the column oven operating at 45°C. Peptides were eluted over a 140 minute gradient ranging from 6 to 60% of 80% acetonitrile, 0.1% formic acid at 250 nl/min, and the Q-Exactive instrument (ThermoFisher Scientific) was run in a DD-MS2 top10 method. Full MS spectra were collected at a resolution of 70,000, with an AGC target of 3×10^6^ or maximum injection time of 20 ms and a scan range of 300–1750 m/z. The MS2 spectra were obtained at a resolution of 17,500, with an AGC target value of 1×10^6^ or maximum injection time of 60 ms, a normalised collision energy of 25 and an intensity threshold of 1.7×10^4^. Dynamic exclusion was set to 60 s, and ions with a charge state <2 or unknown were excluded. MS performance was verified for consistency by running complex cell lysate quality control standards, and chromatography was monitored to check for reproducibility.

**Label-free Quantitative Proteomics Analysis.** The raw files were analysed using Proteome Discoverer 2.4. Label-free quantitation (LFQ) was enabled by the Minora Feature Detector algorithm in the processing step and Precursor Ions Quantifier node in the consensus step, and spectra were matched against the assembled and annotated genome. Dynamic modifications were set as Oxidation (M), Deamidation (N,Q) and Acetyl on protein N-termini. Cysteine carbamidomethyl was set as a static modification. All results were filtered to a 1% FDR. The data was processed using the Perseus software (v. 1.6.15, <https://maxquant.net/perseus/>). Proteins were filtered out if present in less than three of the five replicates of any of the sample. Values were log2-transformed and missing values were imputed from a downshifted normal distribution (width: 0.3, downshift: 1.8). A linear model was applied to the data using LIMMA (7) in Rstudio (v. 3.6.3) and comparisons were made between Δ*tdaB* and WT for each sample fraction (cell or supernatant). To test for significant differences between these comparisons, empirical Bayes statistics were applied to estimate the false discovery rate (FDR, *q*) with a significance threshold of *q*=0.01.

**Extraction of small molecules and metabolome analysis.** Chemical extractions were performed as described by Giubergia *et al*. (8) using 5 mL sample. HR-LCMS was performed on an Agilent Infinity 1290 UHPLC system. Liquid chromatography of 1 µl or 5 µl extract was performed using an Agilent Poroshell 120 phenyl-C6column (2.1 × 150 mm, 1.9 μm) at 60 oC using CH3CN and H2O, both containing 20 mM FA. Initially, a linear gradient of 10% CH3CN/H2O to 100% CH3CN over 10 min was employed, followed by isocratic elution of 100% CH3CN for 2 min. The gradient was returned to 10% CH3CN/H2O in 0.1 min and finally isocratic condition of 10% CH3CN/H2O for 1.9 min, all at a flow rate of 0.35 min ml-1. HRMS data were recorded in positive ionization on an Agilent 6545 QTOF MS equipped with an Agilent Dual Jet Stream electrospray ion (ESI) source with a drying gas temperature of 250°C, drying gas flow of 8 min l-1, sheath gas temperature of 300°C and sheath gas flow of 12 min l-1. Capillary voltage was 4000 V and nozzle voltage was set to 500 V. The HRMS data was processed and analyzed using Agilent MassHunter Qualitative Analysis B.07.00. HPLC grade solvents (VWR Chemicals) were used for extractions while LCMS grade solvents (VWR Chemicals) were used for LCMS. Raw data was converted using MSConvert (ProteoWizard) and preprocessed using both MS-DIAL (9) and MZmine 2.37 (10). All statistical analyses were performed in MetaboAnalyst (11). Molecular networking was all completed within the GNPS platform (12), which includes: Feature Based Molecular Networking (13) and Ion Identity Molecular Networking (14). Ion Identity MN workflow can be found here: <https://gnps.ucsd.edu/ProteoSAFe/status.jsp?task=a685ddf0c0b943d38e7eb95bbb5d4427>. Visualization of the molecular networks was completed using Cytoscape 3.8.2. (15). Molecular formula prediction was completed using SIRIUS (16).

**References**

1. Davis MW, Jorgensen EM. 2022. ApE, A Plasmid Editor: A Freely Available DNA Manipulation and Visualization Program. Front Bioinform 2.

2. Wang H, Li Z, Jia R, Hou Y, Yin J, Bian X, Li A, Müller R, Stewart AF, Fu J, Zhang Y. 2016. RecET direct cloning and Redαβ recombineering of biosynthetic gene clusters, large operons or single genes for heterologous expression. Nat Protoc 11:1175–1190.

3. Zhang S-D, Isbrandt T, Lindqvist LL, Larsen TO, Gram L. 2021. Holomycin, an Antibiotic Secondary Metabolite, Is Required for Biofilm Formation by the Native Producer *Photobacterium galatheae* S2753. Appl Environ Microbiol 87.

4. Liao Y, Smyth GK, Shi W. 2019. The R package Rsubread is easier, faster, cheaper and better for alignment and quantification of RNA sequencing reads. Nucleic Acids Res 47:e47–e47.

5. Love MI, Huber W, Anders S. 2014. Moderated estimation of fold change and dispersion for RNA-seq data with DESeq2. Genome Biol 15:550.

6. Schoof EM, Lechman ER, Dick JE. 2016. Global proteomics dataset of miR-126 overexpression in acute myeloid leukemia. Data Br 9:57–61.

7. Ritchie ME, Phipson B, Wu D, Hu Y, Law CW, Shi W, Smyth GK. 2015. limma powers differential expression analyses for RNA-sequencing and microarray studies. Nucleic Acids Res 43:e47–e47.

8. Giubergia S, Phippen C, Nielsen KF, Gram L. 2017. Growth on Chitin Impacts the Transcriptome and Metabolite Profiles of Antibiotic-Producing *Vibrio coralliilyticus* S2052 and *Photobacterium galatheae* S2753. mSystems 2:1–12.

9. Tsugawa H, Cajka T, Kind T, Ma Y, Higgins B, Ikeda K, Kanazawa M, VanderGheynst J, Fiehn O, Arita M. 2015. MS-DIAL: data-independent MS/MS deconvolution for comprehensive metabolome analysis. Nat Methods 12:523–526.

10. Pluskal T, Castillo S, Villar-Briones A, Orešič M. 2010. MZmine 2: Modular framework for processing, visualizing, and analyzing mass spectrometry-based molecular profile data. BMC Bioinform 11:395.

11. Pang Z, Chong J, Zhou G, de Lima Morais DA, Chang L, Barrette M, Gauthier C, Jacques P-É, Li S, Xia J. 2021. MetaboAnalyst 5.0: narrowing the gap between raw spectra and functional insights. Nucleic Acids Res 49:W388–W396.

12. Wang M, Carver JJ, Phelan V v, Sanchez LM, Garg N, Peng Y, Nguyen DD, Watrous J, Kapono CA, Luzzatto-Knaan T, Porto C, Bouslimani A, Melnik A v, Meehan MJ, Liu W-T, Crüsemann M, Boudreau PD, Esquenazi E, Sandoval-Calderón M, Kersten RD, Pace LA, Quinn RA, Duncan KR, Hsu C-C, Floros DJ, Gavilan RG, Kleigrewe K, Northen T, Dutton RJ, Parrot D, Carlson EE, Aigle B, Michelsen CF, Jelsbak L, Sohlenkamp C, Pevzner P, Edlund A, McLean J, Piel J, Murphy BT, Gerwick L, Liaw C-C, Yang Y-L, Humpf H-U, Maansson M, Keyzers RA, Sims AC, Johnson AR, Sidebottom AM, Sedio BE, Klitgaard A, Larson CB, Boya P CA, Torres-Mendoza D, Gonzalez DJ, Silva DB, Marques LM, Demarque DP, Pociute E, O’Neill EC, Briand E, Helfrich EJN, Granatosky EA, Glukhov E, Ryffel F, Houson H, Mohimani H, Kharbush JJ, Zeng Y, Vorholt JA, Kurita KL, Charusanti P, McPhail KL, Nielsen KF, Vuong L, Elfeki M, Traxler MF, Engene N, Koyama N, Vining OB, Baric R, Silva RR, Mascuch SJ, Tomasi S, Jenkins S, Macherla V, Hoffman T, Agarwal V, Williams PG, Dai J, Neupane R, Gurr J, Rodríguez AMC, Lamsa A, Zhang C, Dorrestein K, Duggan BM, Almaliti J, Allard P-M, Phapale P, Nothias L-F, Alexandrov T, Litaudon M, Wolfender J-L, Kyle JE, Metz TO, Peryea T, Nguyen D-T, VanLeer D, Shinn P, Jadhav A, Müller R, Waters KM, Shi W, Liu X, Zhang L, Knight R, Jensen PR, Palsson BØ, Pogliano K, Linington RG, Gutiérrez M, Lopes NP, Gerwick WH, Moore BS, Dorrestein PC, Bandeira N. 2016. Sharing and community curation of mass spectrometry data with Global Natural Products Social Molecular Networking. Nat Biotechnol 34:828–837.

13. Nothias L-F, Petras D, Schmid R, Dührkop K, Rainer J, Sarvepalli A, Protsyuk I, Ernst M, Tsugawa H, Fleischauer M, Aicheler F, Aksenov AA, Alka O, Allard P-M, Barsch A, Cachet X, Caraballo-Rodriguez AM, da Silva RR, Dang T, Garg N, Gauglitz JM, Gurevich A, Isaac G, Jarmusch AK, Kameník Z, Kang K bin, Kessler N, Koester I, Korf A, le Gouellec A, Ludwig M, Martin H. C, McCall L-I, McSayles J, Meyer SW, Mohimani H, Morsy M, Moyne O, Neumann S, Neuweger H, Nguyen NH, Nothias-Esposito M, Paolini J, Phelan V v., Pluskal T, Quinn RA, Rogers S, Shrestha B, Tripathi A, van der Hooft JJJ, Vargas F, Weldon KC, Witting M, Yang H, Zhang Z, Zubeil F, Kohlbacher O, Böcker S, Alexandrov T, Bandeira N, Wang M, Dorrestein PC. 2020. Feature-based molecular networking in the GNPS analysis environment. Nat Methods 17:905–908.

14. Schmid R, Petras D, Nothias L-F, Wang M, Aron AT, Jagels A, Tsugawa H, Rainer J, Garcia-Aloy M, Dührkop K, Korf A, Pluskal T, Kameník Z, Jarmusch AK, Caraballo-Rodríguez AM, Weldon KC, Nothias-Esposito M, Aksenov AA, Bauermeister A, Albarracin Orio A, Grundmann CO, Vargas F, Koester I, Gauglitz JM, Gentry EC, Hövelmann Y, Kalinina SA, Pendergraft MA, Panitchpakdi M, Tehan R, le Gouellec A, Aleti G, Mannochio Russo H, Arndt B, Hübner F, Hayen H, Zhi H, Raffatellu M, Prather KA, Aluwihare LI, Böcker S, McPhail KL, Humpf H-U, Karst U, Dorrestein PC. 2021. Ion identity molecular networking for mass spectrometry-based metabolomics in the GNPS environment. Nat Commun 12:3832.

15. Shannon P, Markiel A, Ozier O, Baliga NS, Wang JT, Ramage D, Amin N, Schwikowski B, Ideker T. 2003. Cytoscape: A Software Environment for Integrated Models of Biomolecular Interaction Networks. Genome Res 13:2498–2504.

16. Dührkop K, Fleischauer M, Ludwig M, Aksenov AA, Melnik A v., Meusel M, Dorrestein PC, Rousu J, Böcker S. 2019. SIRIUS 4: a rapid tool for turning tandem mass spectra into metabolite structure information. Nat Methods 16:299–302.

17. Grotkjær T, Bentzon-Tilia M, D’Alvise P, Dourala N, Nielsen KF, Gram L. 2016. Isolation of TDA-producing *Phaeobacter* strains from sea bass larval rearing units and their probiotic effect against pathogenic *Vibrio* spp. in *Artemia* cultures. Syst Appl Microbiol 39:180–188.

18. Ruiz-Ponte C, Cilia V, Lambert C, Nicolas JL. 1998. *Roseobacter gallaeciensis* sp. nov., a new marine bacterium isolated from rearings and collectors of the scallop Pecten maximus. Int J Syst Bacteriol 48:537–542.

19. Wang R, Gallant É, Seyedsayamdost MR. 2016. Investigation of the genetics and biochemistry of roseobacticide production in the Roseobacter clade bacterium Phaeobacter inhibens. mBio 7:1–10.

20. Skov MN, Pedersen K, Larsen JL. 1995. Comparison of pulsed-field gel electrophoresis, ribotyping, and plasmid profiling for typing of Vibrio anguillarum serovar O1. Appl Environ Microbiol 61:1540–1545.

21. Milton DL, O’Toole R, Horstedt P, Wolf-Watz H. 1996. Flagellin A is essential for the virulence of *Vibrio anguillarum*. J Bacteriol 178:1310–1319.

22. Obranić S, Babić F, Maravić-Vlahoviček G. 2013. Improvement of pBBR1MCS plasmids, a very useful series of broad-host-range cloning vectors. Plasmid 70:263–267.
